# Matrix Metalloproteinases and Blood-Brain Barrier Disruption in Acute Ischemic Stroke

**DOI:** 10.3389/fneur.2013.00032

**Published:** 2013-04-03

**Authors:** Shaheen E. Lakhan, Annette Kirchgessner, Deborah Tepper, Aidan Leonard

**Affiliations:** ^1^Biosciences Department, Global Neuroscience Initiative Foundation Beverly Hills, CA, USA; ^2^Neurological Institute, Cleveland Clinic Cleveland, OH, USA; ^3^School of Health and Medical Sciences, Seton Hall University South Orange, NJ, USA

**Keywords:** matrix metalloproteinases, blood-brain barrier, stroke, caveolin-1, reactive oxygen species

## Abstract

Ischemic stroke continues to be one of the most challenging diseases in translational neurology. Tissue plasminogen activator (tPA) remains the only approved treatment for acute ischemic stroke, but its use is limited to the first hours after stroke onset due to an increased risk of hemorrhagic transformation over time resulting in enhanced brain injury. In this review we discuss the role of matrix metalloproteinases (MMPs) in blood-brain barrier (BBB) disruption as a consequence of ischemic stroke. MMP-9 in particular appears to play an important role in tPA-associated hemorrhagic complications. Reactive oxygen species can enhance the effects of tPA on MMP activation through the loss of caveolin-1 (cav-1), a protein encoded in the cav-1 gene that serves as a critical determinant of BBB permeability. This review provides an overview of MMPs’ role in BBB breakdown during acute ischemic stroke. The possible role of MMPs in combination treatment of acute ischemic stroke is also examined.

## Introduction

Stroke is the third leading cause of death in industrialized countries (Lo et al., 2003) and the most frequent cause of permanent disability in adults worldwide (Donnan et al., 2008). Acute ischemic stroke is the most common form of stroke and results from sudden blood vessel occlusion by a thrombus or embolism, resulting in an almost immediate loss of oxygen and glucose to the cerebral tissue. Although different mechanisms are involved in the pathogenesis of stroke, increasing evidence shows that ischemic injury and inflammation account for its pathogenic progression (Muir et al., 2007). Cerebral ischemia initiates cascades of pathological events, including vasogenic edema, disruption of the blood-brain barrier (BBB), intracranial hemorrhage (ICH), astroglial activation, and neuronal death. This ultimately causes irreversible neuronal injury in the ischemic core within minutes of the onset (Dimagl et al., 1999).

Despite advances in understanding the pathophysiology of cerebral ischemia, treatment options for acute ischemic stroke remain very limited (Donnan et al., 2008). Intravenous recombinant tissue plasminogen activator (tPA) remains the only FDA-approved thrombolytic therapy for reestablishing blood flow and salvaging brain tissue after acute ischemic stroke (Lijnen and Collen, 1987; National Institute of Neurological Disorders and Stroke rt-PA Stroke Study Group, 1995). By degrading fibrin clots, tPA acts as a thrombolytic agent through the activation of plasminogen to plasmin (Lijnen and Collen, 1987). Although tPA administered within 4.5 h or less of symptom onset improves the functional outcome in patients (Miller et al., 2012; Wardlaw et al., 2012), it induces a 10-fold increase of symptomatic intracranial hemorrhage (ICH) (National Institute of Neurological Disorders and Stroke rt-PA Stroke Study Group, 1995). Furthermore, delayed reperfusion with tPA beyond 3 h is associated with an increased risk of hemorrhagic transformation (HT) with enhanced brain injury (Clark et al., 1999). Moreover, tPA may cause injury to the BBB by activating matrix metalloproteinases (MMPs) (Wang et al., 2003). Thus, the therapeutic application of tPA is limited to specific clinical settings (National Institute of Neurological Disorders and Stroke t-PA Stroke Study Group, 1997). There is a pressing need to identify new combination therapies that can prevent tPA-associated ICH as well as extend the time window for thrombolysis without reducing its benefits.

Recent studies suggest that tPA adverse effects are mediated through MMPs, a family of >20 zinc-dependent enzymes that increase BBB permeability by degrading components of the extracellular matrix (ECM) and tight junctions (TJ) in endothelial cells (ECs) (Lapchak et al., 2000; Lijnen, 2001; Briasoulis et al., 2012). Increased expression and activation of MMPs plays a pivotal role in thrombolysis-mediated BBB leakage and edema, resulting in intracranial hemorrhage (Lapchak et al., 2000; Sumii and Lo, 2002). Reactive oxygen species (ROS) and its signaling pathways can enhance the effects of tPA on MMP activation (Harada et al., 2012). In this review we provide an overview of the role of MMPs in BBB breakdown during acute ischemic stroke and the potential for MMP inhibition in the treatment of stroke.

## Structural Components of the BBB/Neurovascular Unit

The BBB is a dynamic interface between the peripheral circulation and the CNS. It controls the influx and efflux of biological substances needed for the brain metabolic processes, as well as for neuronal function. Thus, the functional and structural integrity of the BBB is vital in maintaining brain homeostasis. (Cucullo et al., 2011).

The structure of the BBB has been discussed in reviews elsewhere (Sandoval and Witt, 2008; Abbott et al., 2010). Briefly, the anatomical substrate of the BBB is the cerebral microvascular endothelium, which together with the closely associated astrocytes, pericytes, neurons, and the ECM, constitute a “neurovascular unit” that is essential for the health and function of the CNS (Hawkins and Davis, 2005). Cell–cell interactions in the neurovascular unit form the basis for brain function. Dysfunctional signaling in the neurovascular unit underlies the basis for disease. Alterations in microvessel integrity may have other effects within the neurovascular unit that affect neuronal function. The mechanisms of neurovascular unit response to stroke are not fully understood. However, any fully effective stroke therapy must include both prevention of cell death as well as repair of integrated neurovascular function.

The microcapillary endothelium is composed of TJs and adherens junctions (AJs). Both TJs and AJs act to restrict permeability across the endothelium (Bazzoni and Dejana, 2004). TJs are continuous membrane strands located at the apical site between brain ECs, which consist of transmembrane proteins (junctional adhesion molecule-1, claudins, and occludin), and cytoplasmic proteins (zonula occludens-1 to -3) linked to the actin cytoskeleton (Morita et al., 1999). Among the claudin family members, claudin-5 has been shown to be a major cell adhesion molecule of BBB TJs (Yang and Rosenberg, 2011). The structure and function of TJ proteins can be regulated by alternating their expression and/or distribution at the BBB during ischemic stroke. Phosphorylation is a major regulatory mechanism of both transmembrane and accessory proteins at the TJ (Staddon et al., 1995; Stuart and Nigam, 1995; Sakakibara et al., 1997; Yamamoto et al., 2008). Disruption of BBB TJ by disease or drugs can lead to impaired BBB function, compromising CNS function (Hawkins and Davis, 2005). Therefore, understanding exactly how BBB TJ might be vulnerable to damage may lead to precise, effective prevention and treatment of neurological diseases.

Adherens junctions are ubiquitous in the vasculature, mediating the adhesion of ECs to each other. The AJs are mainly composed of vascular endothelial VE-cadherin, a Ca^2+^-regulated protein that facilitates cell to cell adhesion by forming a continuous belt near the apical end of the junctional cleft (Vincent et al., 2004). Although disruption of AJs at the BBB can lead to increased permeability, it is primarily the TJ that confers low paracellular permeability and high electrical resistance (Abbruscato and Davis, 1999). AJs may play an important role in the localization and stabilization of the TJs. Endothelial VE-cadherin upregulates TJ protein claudin-5, suggesting a direct regulation of TJ integrity by AJ proteins (Taddei et al., 2008).

## Matrix Metalloproteinases

Matrix metalloproteinases, also called matrixins, comprise a family of enzymes that cleave protein substrates based on a conserved mechanism involving activation of a site-bound water molecule by a Zn^2+^ ion. MMPs consist of 23 distinct proteases in humans (24 in mouse). Excreted MMPs are generally classified according to their substrate specificity, leading to four classes: the collagenases (MMP-1, -8, and -13), the gelatinases (MMP-2 and -9), the stromelysins (MMP-3, -10, and -11) and a heterogenous group containing matrilysin (MMP-7), metallo-elastase (MMP-12), enamelysin (MMP-20), endometase (MMP-26), and epilysin (MMP-28) (Klein and Bischoff, 2011). An alternate classification arranges the MMPs according to their domain structure (Sternlicht and Werb, 2001). Most of the MMPs are synthesized as inactive latent enzymes. Conversion to the active enzyme is generally mediated by activator systems that include plasminogen activator or the pro-hormone convertase, furin (Malemud, 2006). Although the catalytic domain of MMPs is structurally similar, there are many differences in substrate specificity, cellular and tissue localization, membrane binding and regulation, making this a versatile family of enzymes with a multitude of physiological functions, which are not fully understood. Essentially, all members of the MMP family have been linked to disease development, notably to cancer metastasis, chronic inflammation with ensuing tissue damage, as well as to neurological disorders (for review see Klein and Bischoff, 2011).

**Figure 1:**
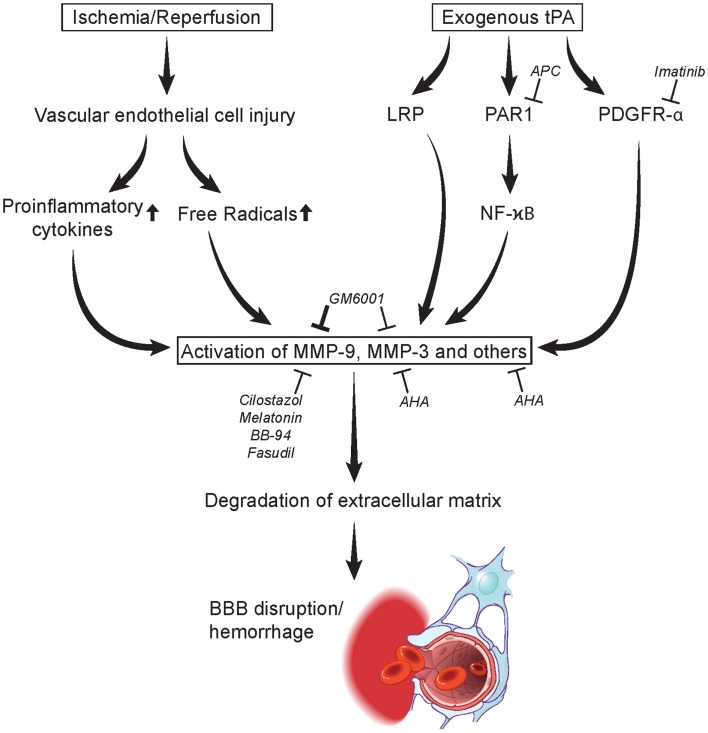
**Mechanisms in MMP activation leading to degradation of extracellular matrix and blood-brain barrier disruption/hemorrhage**. Acute stroke may lead to vascular endothelial cell injury causing the release of proinflammatory cytokines and free radicals at the neurovascular unit activating MMPs. tPA administration may likewise induce MMPs through the LRP, PAR1, and PDGRF-α pathways. MMP activation is inhibited by several agents including imatinib, activation protein C (APC), cilostazol, melatonin, BB-94, GM6001, fasudil, AHA, NYX-059, and edaravone (Table 2). LRP, lipoprotein receptor-related protein; MMP, matrix metalloproteinase; PAR1, protease-activated receptor 1; tPA, tissue type plasminogen activator. Figure modified from Yamashita and Abe (2011).

Matrix metalloproteinase activity is regulated by a group of endogenous proteins called tissue inhibitor of metalloproteinases (TIMPs), which bind to active and alternative sites of activated MMP (Malemud, 2006). Four homologous TIMPs (TIMPs 1–4) have been identified to date (21–30 kDa in size) (Gomez et al., 1997). In a combined laser microdissection and protein array study, TIMP-1 was upregulated in the infarcted tissue in human brain after stroke compared to healthy controls (Cuadrado et al., 2009). In a mouse model of focal cerebral ischemia (30 min of middle cerebral artery occlusion, MCAO), the deletion of the TIMP-1 gene resulted in increased MMP-9 protein expression and gelatinolytic activity, and was accompanied by exacerbated BBB disruption, neuronal apoptosis, and ischemic injury when compared to wild-type (WT) animals (Fujimoto et al., 2008). Interestingly, TIMP-2 knockout (KO) mice also had a leakier BBB compared to WT animals, but no increase in MMP-9 expression or exacerbation of neuronal loss when compared to WT mice (Fujimoto et al., 2008). By contrast, when transgenic mice overexpressing the human TIMP-1 gene under the control of the metallothionein-1 promoter were subjected to controlled cortical impact, reduced 24-h MMP-9 levels and a less leaky BBB, as well as decreased brain tissue damage at 7 days post-trauma were observed compared to control animals (Tejima et al., 2009). In a model of transient 2-h focal cerebral ischemia, MMP-9 levels were lower in TIMP-1 KO mice compared to WTs. Correspondingly, BBB leakage was ameliorated by TIMP-1 overexpression, and 24-h infarction volumes were also reduced. These observations collectively suggest that MMPs could represent the potential targets for pharmacological intervention in stroke and the inhibition of their activity may actually have therapeutic effects (Figure 1) (Maier et al., 2006).

The injured brain has various cell types that can express MMPs, including resident and infiltrating inflammatory cells. However, the brain regions and cellular sources of expression differ for the specific MMPs, as well as the type, severity, and duration of injuries (Cheng et al., 2006). MMPs can also be released from invading leukocytes (Chou et al., 2011). In particular, MMP-8 is known as the “neutrophil collagenase” (Hasty et al., 1986).

### Role in BBB disruption

Disruption of the BBB during focal cerebral ischemia/reperfusion injury has long been considered to follow a biphasic time course. Morphologically, BBB opening correlates with a redistribution of the TJ and AJ proteins from the plasma membrane to the cytoplasm as well as reorganization of the endothelial actin cytoskeleton. The extent of BBB disruption is associated with the type, severity, and duration of ischemic insults. The molecular mechanisms underlying BBB opening are not fully understood, although several MMPs are believed to regulate BBB permeability and function during ischemic stroke (Mun-Bryce and Rosenberg, 1998).

The expression of MMPs in the adult brain is very low to undetectable, but clinical and experimental studies have shown that several MMPs are upregulated and activated after ischemic stroke (Lee et al., 2007; McColl et al., 2008). MMPs disrupt the BBB by degrading the TJ proteins and basal lamina proteins, thereby leading to BBB leakage, leukocyte infiltration, brain edema, and hemorrhage. Evidence suggests that MMP-2 and MMP-9 play different roles in BBB disruption during ischemic stroke. MMP-2 KO does not provide neuroprotection in mouse models of permanent and transient MCAO (Asahi et al., 2001b). Consistently, *in vitro* data show that MMP-2 is not toxic to neurons in hippocampal slice preparations (Cunningham, 2005). In contrast, MMP-9 KO provides strong neuroprotection in the same animal models, and *in vitro* MMP-9 is toxic to neurons in hippocampal slice preparations and in cultured primary cortical neurons (Asahi et al., 2000b). In support of these data, a clinical study (Lucivero et al., 2007) reported an increase in plasma MMP-2 only in patients with lacunar (mild) stroke early (within 12 h) and this was related to better outcome. In contrast, an increase in plasma MMP-9 was observed later (at day 7) and related to more severe stroke.

Matrix metalloproteinases are thought to have beneficial roles in stroke recovery. Shortly after an ischemic insult, a cascade of events is initiated in an attempt to repair the damage, a process similar to that found in wound healing (National Institute of Neurological Disorders and Stroke rt-PA Stroke Study Group, 1995; Wardlaw et al., 2012). Following injury, blood vessels are dependent on the plasminogen activator system and on MMPs for their regeneration (Suzuki et al., 2009). It may be that a balanced level of MMP activity is important for vascular remodeling after ischemic brain injury (Yang and Rosenberg, 2011). Therefore, extended inhibition of MMPs, especially through the use of broad-spectrum inhibitors, might prove deleterious (National Institute of Neurological Disorders and Stroke rt-PA Stroke Study Group, 1995; Donnan et al., 2008).

### MMP-2 (gelatinase A)

Matrix metalloproteinase-2 is one of the two described human gelatinases in the MMP family, named for their ability to proteolytically degrade gelatine (denatured collagen) (see Table 1 for a list of MMPs and their putative roles in acute ischemic stroke). MMP-2 is ubiquitously expressed as a 72-kDa proenzyme and subject to extensive glycosylation (Klein and Bischoff, 2011).

**Table 1:** **MMPs and their putative role in acute ischemic stroke**.

MMP	Putative role
MMP-1 (collagenase-1)	Preferentially cleaves type III collagen. Upregulated in infracted tissue. Limited studies
MMP-2 (gelatinase A)	Attacks major components of the basal lamina around the cerebral blood vessels including type IV collagen, laminin, and fibronectin. May contribute to infarction and hemorrhagic volume
MMP-3 (stromelysin-1)	Degrades the extracellular matrix proteins fibronectin, denatured collagen, laminin, and proteoglycans. Plays a key role in the initial opening of the BBB after stroke and the development of hemorrhagic transformation, particularly with tPA treatment
MMP-8 (collagenase-2)	Preferentially cleaves type I collagen. Similar to MMP-1, is upregulated in infracted tissue and studies are limited
MMP-9 (gelatinase B)	Attacks major components of the basal lamina including type IV collagen, laminin, and fibronectin. Plays a key role in the delayed opening of the BBB after stroke especially in states of systematic inflammation. Also implicated in the development of hemorrhagic transformation particularly with tPA treatment
MMP-10 (stromelysin-2)	Disables thrombin-activatable fibrinolysis inhibitor, thereby enhancing tPA-induced fibrinolysis. May hasten reperfusion time and limit infarction volume
MMP-13 (collagenase-3)	Preferentially cleaves type II collagen. Functions may mirror MMP-9

*BBB, blood-brain barrier; MMP, matrix metalloproteinase; tPA, tissue plasminogen activator*.

A study provided indirect evidence that MMP-2 played a key role in initial opening of the BBB after cerebral ischemia (Rosenberg et al., 1998). In a rat model of transient MCAO, the initial opening of the BBB occurred as early as 3 h after reperfusion and increased activation of MMP-2 correlated with the early opening of the BBB and the degradation of the TJ proteins claudin-5 and occludin in both cerebral hemispheres (Rosenberg et al., 1992; Yang et al., 2007). Experimental data clearly demonstrated an increase in MMP-2 at 3 h, along with increased expression of the MMP-2 activators, MT1-MMP and furin. A synthetic MMP inhibitor (BB-1101) blocked the increase in brain MMP-2 levels, but it did not have any effect on stroke lesion size at 48 h after MCAO and had significant adverse effects on neurologic function in rats at 3 and 4 weeks after MCAO (Rosenberg et al., 1992, 1998; Yang et al., 2007). In contrast, direct injection of MMP-2 into the rat brain resulted in the disruption of the BBB with subsequent hemorrhage, and this effect was inhibited by co-administration of TIMP-2 (Rosenberg et al., 1992). Thus, the early degradation of TJ proteins seems to be associated with a marked increase in MMP-2 in the early phase of ischemia.

Suofu et al. (2012) recently assessed the effects of MMP-2 KO, MMP-9 KO, and MMP-2/9 double KO (dKO) in protecting against mechanical reperfusion-induced HT and other brain injuries after the early stages of cerebral ischemia in mice of the same genetic background. Both MMP-2 and MMP-9 specifically attack the type IV collagen, laminin, and fibronectin, which are the major components of the basal lamina around the cerebral blood vessels. MCAO was performed and reperfusion was started at 1 or 1.5 h after onset of MCAO. Mice were sacrificed 8 h later. Both pro- and active-MMP-2 and MMP-9 levels were significantly elevated in the early ischemic brain. After the early stages of ischemia and reperfusion, the hemorrhagic incidence was reduced in the cortex of MMP-2 KO mice. The hemorrhagic volume was also significantly decreased in the cortexes of MMP-2 and/or -9 KO mice. In the basal ganglia, MMP-2 KO and MMP-2/9 dKO mice displayed a remarkable decrease in hemorrhagic volume, but MMP-9 deletion did not protect against hemorrhage. MMP-2 and/or -9 KO mice displayed significantly decreased infarction volume in both the cortex and striatum, in addition to improved neurological function. The results suggested that MMP-2 deficiency as well as MMP-2 and MMP-9 double deficiency were more protective than MMP-9 deficiency alone against HT after the early stages of ischemia and reperfusion.

### MMP-3 (stromelysin-1)

Matrix metalloproteinase-3 (stromelysin-1) was first described in 1985 as a 51-kDa protein secreted by rabbit fibroblasts (Chin et al., 1985). MMP-3 could be distinguished from collagenases by the inability to degrade type I collagen. The substrate specificity of MMP-3 is broad and MMP-3 has been found to degrade many ECM proteins including fibronectin, denatured collagens (gelatin), laminin, and proteoglycans (Klein and Bischoff, 2011).

Matrix metalloproteinase-3 appears to play an important role in ICH induced by tPA treatment of ischemic stroke in mice. Using the MCAO model, Suzuki et al. (2007) demonstrated that deficiency of the *stromelysin-1* (*MMP-3*) gene, but not the *MMP-9* gene, in mice reduced the increased risk of ICH caused by tPA treatment. In addition, administration of the broad-spectrum MMP inhibitor GM6001 after tPA treatment significantly reduced ICH in WT, but had no effect in MMP-3-deficient mice (Table 2). Furthermore, using a mouse stroke model, tPA treatment induced MMP-3 expression selectively in ECs damaged by ischemia. This suggests that MMP-3 might be involved in degradation of the blood vessel barrier and contribute to ICH. Although MMP-9 expression was also significantly increased at the ischemic areas of the brain, the amount and the distribution were comparable in mice with and without tPA treatment. These data with gene-deficient mice suggest that MMP-3 is relatively more important than MMP-9 for the increased ICH induced by tPA treatment of ischemic stroke in mice. In a combined laser microdissection and protein array study, Cuadrado et al. (2009) found MMP-3 upregulated in the infarcted tissue in human brain after stroke, along with several other MMPs and TIMP-2. MMP-9 and TIMP-2 were amplified in brain microvessels while MMP-10 was notably increased in neurons of the ischemic brain but not in healthy areas. MMP-3 gene polymorphisms were associated with ischemic stroke but not ICH in the Korean population (Kim et al., 2012).

**Table 2:** **Drugs that can attenuate tPA-related hemorrhagic transformation of ischemic stroke**.

Drug	Proposed mechanism	Model and reference
Imatinib (PDGFR-α antagonist)	Inhibits PDGFR-α activity	Mouse MCAO (Su et al., 2008)
Activated protein C (APC)	Inhibits the tPA-PAR1-MMP-9 pathway	Mouse and Rat MCAO (Cheng et al., 2006)
Cilostazol (phosphodiesterase III inhibitor)	Inhibits MMP-9 activity	Mouse MCAO (Ishiguro et al., 2010)
		Rat MCAO (Choi et al., 2002)
		Human trial (Lee et al., 2003)
		Mouse MCAO (Nonaka et al., 2009)
Melatonin	Inhibits MMP-9 activity	Mouse MCAO (Chen et al., 2006)
BB-94 (MMP-9 inhibitor)	Inhibits MMP-9 activity	Rat MCAO (Pfefferkorn and Rosenberg, 2003)
Fasudil (rho kinase inhibitor)	Inhibits MMP-9 activity	Mouse MCAO (Ishiguro et al., 2012)
Minocycline (broad-spectrum tetracycline antibiotic)	Inhibits MMP-9 activity	Human trial (Switzer et al., 2011)
		Human trial (Fagan et al., 2010)
GM6001	Inhibits MMP-3 > MMP-9 activity	Mouse MCAO (Suzuki et al., 2007)
AHA (*p*-aminobenzoyl-gly-pro-d-leu-d-ala-hydroxamate; broad-spectrum MMP inhibitor)	Broad-spectrum MMP inhibitor	Rat MCAO (Copin et al., 2011)
NXY-059	Scavenges free radicals	Rat MCAO (Lapchak et al., 2002)
		Rat MCAO (Green and Ashwood, 2005)
Edaravone	Scavenges free radical	Rat MCAO (Yamashita et al., 2009)

*MCAO, middle cerebral artery occlusion; MMP, matrix metalloproteinase; tPA, tissue plasminogen activator*.

The mechanism underlying MMP-3 induction by tPA appears to involve the low-density lipoprotein receptor-related protein (LRP)/nuclear factor kappa-B (NF-κB) pathway. LRP, a member of the lipoprotein receptor family, is a scavenger receptor that binds a variety of biologic ligands, including tPA (Herz and Strickland, 2001). In the brain, LRP is found in neurons and perivascular astrocytes (Polavarapu et al., 2007). *In vitro*, tPA induces MMP-3 in cultured murine brain ECs, and this effect is prevented by inhibition of either LRP or the NF-κB activation (Suzuki et al., 2009). The involvement of the LRP/NF-κB pathway was also reported in the induction of MMP-3 by tPA under ischemic stress. In addition, the increase in both ICH and the induction of MMP-3 by tPA was suppressed by treatment with RAP, suggesting that ICH caused by tPA could be suppressed by LRP inhibition. LRP production is reported to be upregulated in ECs exposed to ischemia, and elevated LRP levels have been implicated in the increased ICH risk associated with delayed tPA treatment. This implies that the tPA/LRP/MMP-3 pathway may be a suitable target for developing strategies to improve the therapeutic efficacy of tPA in acute ischemic stroke.

Matrix metalloproteinase-3 (stromelysin-1) has also been shown to mediate BBB opening during neuroinflammation (Gurney et al., 2006). After intracerebral injection of lipopolysaccharide (LPS), *MMP-3* KO mice showed less degradation of the TJ proteins (claudin-5, occludin, laminin-alpha-1) together with reduced neutrophil infiltration when compared with WT mice (Gurney et al., 2006). In the rat transient MCAO model, brain MMP-3 is activated as determined by the cleavage of the cerebral matrix agrin, an MMP-3 substrate (Sole et al., 2004).

### MMP-9 (gelatinase B)

Matrix metalloproteinase-9 (gelatinase B), first described in neutrophils in 1974 (Sopata and Dancewicz, 1974), is expressed as a 92-kDa proenzyme, which can be activated to the 83-kDa mature enzyme (Klein and Bischoff, 2011). Among MMPs, MMP-9 is the most widely studied enzyme in acute ischemic stroke. In particular, MMP-9 activity is significantly elevated in human brain tissue and serum after stroke (Clark et al., 1997; Montaner et al., 2001; Horstmann et al., 2003; Switzer et al., 2011) as well as in animal stroke models beginning at 12 h after permanent MCAO [54]. High plasma MMP-9 concentrations in the acute phase of a cerebral infarction is considered to be independent predictor of HT in all stroke subtypes (Planas, 2001; Castellanos et al., 2003). MMP-9 has been shown to degrade TJ proteins (claudin-5, occludin, ZO-1) in cultured brain ECs (Chen et al., 2009) and in animal models of focal cerebral ischemia (Yang et al., 2007; McColl et al., 2008). Aberrant MMP-9 proteolytic activity degrades not only TJ proteins but also basal membrane proteins (e.g., fibronectin, laminin, collagen, and others). This degradation is associated with an in increase in BBB permeability, resulting in brain infarction, edema, and HT in both animal models (Lee et al., 2007; Rosenberg and Yang, 2007; Yang et al., 2007) and in human stroke patients (Gasche et al., 1999; Castellanos et al., 2003; Lo, 2008). Moreover, cerebral infarct size is reduced in mice deficient in MMP-9 or after treatment with MMP inhibitor; this effect was associated with reduced degradation of the MMP-9 substrate ZO-1 as compared to WT mice (Asahi et al., 2000b, 2001a; Jiang et al., 2001). Early (day 1) MMP-9 inhibition reduced infarction of day 14; however, benefit was lost when the treatment was delayed until day 3. Stroke pathology was exacerbated when administration was delayed until day 7 (Lo, 2008).

In contrast to MMP-2, which plays a key role in the initial opening of the BBB after cerebral ischemia (see above), experimental studies suggest that MMP-9 appears to be more important in the second, delayed opening of the BBB after ischemic stroke (Adibhatla and Hatcher, 2008). In a rat model of transient MCAO, the initial opening at 3 h correlated with increased brain MMP-2 levels. In contrast, the second, delayed opening (maximal opening at 48 h) appeared to correlate with brain MMP-9 levels (maximally elevated at 48 h) (Rosenberg et al., 1998). An increase in plasma MMP-9 appears later, at day 7, and is correlated to more severe patient strokes (Lucivero et al., 2007).

There is controversy regarding timing of brain MMP-9 expression and activation after ischemic stroke. In the rat transient MCAO model, Planas (2001) show that MMP-9 is induced and activated from 4 h to 4 days. In the mouse permanent MCAO model, Gasche et al. (1999) show induced expression and activation of MMP-9 in the ischemic brain with increased permeability as early as 2–4 h after ischemia. More recently, Demir et al. (2012) reported that plasma MMP-9 levels substantially increased during the acute period of ischemic stroke and were correlated with disease severity and infarct volume in patients with stroke. These studies suggest that MMP inhibition could have a beneficial effect on the outcome of stroke but the effect will depend on the timing of treatment to the stage of brain injury (Lo, 2008).

A recent review of the literature concerning MMP-9 and stroke suggests that MMP-9 is a possible marker for acute ischemic stroke (Ramos-Fernandez et al., 2011). This review revealed that higher MMP-9 values were significantly correlated with larger infarct volume, severity of stroke, and worse functional outcome. There were significant differences in MMP-9 levels between patients with acute ischemic stroke and healthy control subjects. Moreover, MMP-9 was a predictor for the development of ICH in patients treated with thrombolytic therapy. MMP-9 level was significantly increased after stroke onset, with the level correlating with infarct volume, stroke severity, and functional outcome (Ramos-Fernandez et al., 2011). Clinical studies have shown a correlation between plasma MMP-9 levels and the rate of HT in human stroke (Montaner et al., 2001; Castellanos et al., 2007). Montaner et al. (2003) reported that a high level of plasma MMP-9 expression in stroke patients could predict HT after thrombolysis. Recently, Demir et al. (2012) reported that plasma MMP-9 level substantially increased during the acute period of ischemic stroke and correlated with the severity of the disease and infarct volume in patients with acute ischemic stroke. A substrate of MMP-9, fibronectin, has also been reported to be a useful biomarker for predicting HT (Castellanos et al., 2007).

Defining the precise role of plasma MMP-9 after ischemic stroke will have important diagnostic implications for stroke and help in the development of therapeutic strategies aimed at modulating plasma MMP-9. Recently, it was shown that MMP-9 mRNA concentration was almost three times higher in non-survival patients compared to survival patients with acute stroke (Graham et al., 2012); therefore, MMP-9 mRNA was a predictor of poor outcome and mortality in stroke. Nevertheless, there is no significant association between MMP-9 genetic variations or MMP-9 expression and HT occurrence after tPA treatment in stroke patients (Fernandez-Cadenas et al., 2012).

Although MMP-9 is detectable in the brain after stroke, its cellular source remains controversial. Endothelium, glia, and neurons have been shown to display MMP-9 immunoreactivity after ischemia. However, since MMP-9 functions as a protease after being secreted from cells, the location of MMP-immunoreactivity does not necessarily reflect the cells releasing MMP-9 (Wang et al., 2009). Experimental data indicate that brain microvascular ECs (BMECs) and infiltrating leukocytes (most likely neutrophils) are key cellular sources of brain MMP-9 at least in the early phase (within 24 h) after focal cerebral ischemia (Justicia et al., 2003; McColl et al., 2008; Jin et al., 2010). Gidday et al. (2005) used chimeric mice with MMP-9 KO and WT mice to demonstrate that leukocytes are the major source of MMP-9 in the ischemic brain at 24 h following 2 h MCAO. By immunostaining and microdissection, clinical data confirm that microvessel endothelium and infiltrating neutrophils are the major source of the increased brain MMP-9 after ischemic and hemorrhagic stroke in humans (Rosell et al., 2006). In contrast, Harris et al. (2005) failed to detect an impact on MMP-9 levels from neutrophil depletion in ischemic rat brain at 24 h after 3-h MCAO. Thus, infiltrating leukocytes may not be the sole source of MMP-9 in the ischemic brain at later reperfusion times.

There are temporal and spatial changes of MMP-9 within the cells of the neurovascular unit after stroke. In a rat model of transient MCAO, most of the MMP-9 activities co-localized with brain microvessel ECs within 24 h, but at 7–14 days the MMP-9 signal shifted to the periphery of the cortical infarction and was mainly associated with neurons and astrocytes (Zhao et al., 2006). This redistribution likely reflects multiphasic roles of MMP-9 in ischemic stroke. Inhibition of MMP-9 during the late phase (7–14 days) after stroke has been shown to reduce the number of neurons and new vessels, and that correlated with increased brain injury and impaired functional recovery. Thus, it has been noted that blocking MMPs at a badly chosen time in non-target cell types may result in unwanted side effects (Zlokovic, 2006).

In several peripheral organs, MMP-9 derived from bone marrow-derived cells (BMDC) is functionally significant (Wang et al., 2009). BDMC have also been suggested to be an important source of MMP-9 in the brain after ischemia (Gidday et al., 2005; McColl et al., 2008). Results from experiments using bone marrow chimera indicate that BMDC are the major cellular source of MMP-9 detectable in the ischemic brain after 90 min of MCAO and 1 h of reperfusion, which is consistent with previous findings. In addition, MMP-9 released from BMDC contributes to BBB dysfunction at 1 h after reperfusion and subsequent infarct formation at 24 h.

There is evidence indicating that specific inhibition of MMP activity during stroke onset or immediately following brain injury improves neurological outcomes (Table 2). Murata et al. reported that minocycline, a broad-spectrum MMP inhibitor, could reduce neuronal cell death after ischemia and extend the thrombolytic time window (Murata et al., 2008). Moreover, MMP-9 KO can stabilize the BBB by preventing degradation of the TJ protein ZO-1 (Asahi et al., 2001a). A recent clinical trial indicates that minocycline can lower plasma MMP-9 levels, even at 72 h after stroke, and improve neurological outcomes in acute ischemic stroke patients treated with tPA (Switzer et al., 2011).

Cui et al. (2012) recently used an embolic cerebral ischemia mouse model to evaluate (4-phenoxyphenylsulfonyl)methylthiirane (referred to as SB-3CT), the first mechanism-based selective and potent MMP inhibitor selective for gelatinases (Brown et al., 2000). Active gelatinases bind to SB-3CT and catalyze the opening of the thiirane ring in the molecule. The resultant species generated within the active site of the enzyme affords tight binding between the inhibitor and the enzyme. SB-3CT prevents proteolysis of the ECM basement membrane (BM) laminin and protects neurons from focal cerebral ischemia (Gu et al., 2005). Most importantly, significant therapeutic benefit can be observed for up to 6 h after initial injury. Cui et al. demonstrated MMP-9 activation and neurovasculature impairment in stroke and showed the ability of SB-3CT to inhibit MMP-9 activity *in vivo*, which resulted in maintenance of laminin, antagonism of pericyte contraction and loss, and preservation of laminin-positive pericytes and ECs. Repeated dose administration of SB-3CT for 7 days offered beneficial results. In contrast, delayed treatment with the broad-spectrum MMP inhibitors FN-439 (day 3 or 7) and BB-94 (day 7) significantly worsened infarct volumes (Zhao et al., 2006). Thus, SB-3CT rescued neurons from apoptosis and prevented ICH. Selective targeting of MMP-9 antagonizes disruption of the neurovascular unit and protects against brain damage and vessel constriction. Taken together, these results strongly indicate that the thiirane class of gelatinase selective inhibitors holds great promise as therapeutic agents for the treatment of stroke.

Although MMP-9 inhibition or KO can attenuate proteolysis of BBB (Lijnen, 2001; Harada et al., 2012), more recent studies suggest its possible role in neurovascular regeneration, especially in the delayed phase of cerebral ischemia (Bazzoni and Dejana, 2004). Following injury, blood vessels are dependent on the plasminogen activator system and on MMPs for their regeneration. For example, Zhao et al. (2006) showed that delayed inhibition of MMPs by broad-spectrum inhibitors is detrimental during the ischemic recovery stage 7 days after cerebral ischemia, diminishing brain repair in mice, and attenuating neurovascular regeneration in the penumbra. Thus, successful anti-stroke therapies require selective inhibition of aberrant activity without altering the physiological function of MMPs, such as their roles in axonal growth, synaptic plasticity, and vascular remodeling following stroke (Mun-Bryce and Ga, 1998; Malemud, 2006; Adibhatla and Hatcher, 2008; Taddei et al., 2008; Yang and Rosenberg, 2011).

Several studies suggest MMP-9 may play a more prominent role in BBB disruption during ischemic stroke under clinical conditions linked to elevated systemic inflammation. Experimental data have shown that systemic inflammation increases neutrophil infiltration into the ischemic brain, thus altering the kinetics of the BBB TJ disruption after experimental stroke (McColl et al., 2008). These studies demonstrate that infiltrating neutrophils are the primary source of increased (fivefold) MMP-9 activity in the ischemic brain of mice challenged with interleukin-1β (IL-1β) at 4, 8, or 24 h after focal cerebral ischemia. A transformation from transient to sustained BBB disruption caused by enhanced neutrophil-derived MMP-9 is a critical mechanism underlying the exacerbation of ischemic brain injury by IL-1β-induced systemic inflammation. This inflammatory state results in a sustained disruption of the TJ protein (claudin-5) and enhanced disruption of the cerebrovascular basal lamina protein (collagen-IV) (McColl et al., 2008). The poor clinical outcome in stroke patients with elevated systemic inflammatory status may stem from this mechanism.

### MMP-10 (stromelysin-2)

In addition to MMP-2/-3/-9, MMP-10 (stromelysin-2) may also play an active role in mediating BBB disruption during ischemic stroke. MMP-10, which has 82% sequence homology with MMP-3, is secreted as a 53-kDa proenzyme and is activated to a 47-kDa mature protease. The physiological function of MMP-10 is poorly understood, with only a handful of publications attempting characterization of this protease. MMP-10 has been linked to inflammatory/thrombotic processes and vascular integrity, but whether MMP-10 could have a profibrinolytic effect and represent a promising thrombolytic agent is unknown. The effect of MMP-10 on fibrinolysis was studied *in vitro* and *in vivo* in MMP-10 KO mice using two different murine models of arterial thrombosis, laser-induced carotid injury and ischemic stroke (Orbe et al., 2011). *In vitro*, it was shown that MMP-10 was capable of enhancing tPA-induced fibrinolysis. This was done via a mechanism that disabled the thrombin-activatable fibrinolysis inhibitor. *In vivo*, active recombinant human MMP-10 reversed delayed fibrinolysis after photochemical carotid injury in MMP-10 KO mice. In a thrombin-induced stroke model, the reperfusion and infarct size in sham or tPA-treated animals were much worse in MMP-10 KO mice. In this model, administration of active MMP-10 to WT animals significantly reduced blood reperfusion time and infarct size to the same extent as tPA and was associated with shorter bleeding time and no intracranial hemorrhage. This benefit was not seen in thrombin-activatable fibrinolysis inhibitor-deficient mice, suggesting thrombin-activatable fibrinolysis inhibitor inactivation as one of the mechanisms involved in the MMP-10 profibrinolytic effect.

### MMP-13 (collagenase-3) and others

Matrix metalloproteinase-13 (collagenase-3) is the latest human collagenase described in the literature. This enzyme exhibits preference toward cleavage of type II collagen, effectively completing the substrate spectrum of the collagenases. Collagenase-3 was first cloned from breast cancer tissue in 1994 (Freije et al., 1994). This MMP has been thoroughly studied in aggressive cancer as a biomarker of tumor progression (Balbin et al., 1999). In all cases, high expression of MMP-13 seems to be related to the aggressiveness of the tumor. In addition, MMP-13 plays an important role in bone turnover, and this enzyme has been linked to various bone-related diseases (Burrage et al., 2006), making selective MMP-13 inhibitors attractive therapeutic compounds. MMP-13 has also been shown to be involved in tissue injury after stroke (Heim-Riether et al., 2009). Only one earlier study has attempted to measure MMP-13 in human stroke within the first 12 h of symptoms as compared with a healthy control group, but no difference was found (Horstmann et al., 2003). In contrast, Rosell et al. (2005) investigated the blood levels of several MMPs in stroke patients with MCAO who received thrombolytic therapy. High levels of both MMP-9 and MMP-13 were detected in the hyperacute phase of stroke and were correlated with an increase in diffusion-weighted imaging lesions within the first 24 h. A positive correlation was also found between these two metalloproteinases. Thus, both MMP-9 and MMP-13 are involved in tissue injury and cell death, counteracting the benefits of thrombolytic therapy.

The role of other MMPs in stroke are not well described although it also known that MMP-1 (collagenase-1) and MMP-8 (neutrophil collagenase or collagenase-2) are upregulated in the infarcted tissue in human brain compared to healthy control areas (Cuadrado et al., 2009). MMP-8 is very similar to MMP-1 in structure and physiological function, although subtle differences in substrate selectivity exist. MMP-8 has a stronger affinity toward type I collagen than MMP-1, while MMP-1 preferentially cleaves types III collagen (Balbin et al., 1998). Additional studies examining the role of MMP-1 and MMP-8, and other MMPs in stroke are clearly warranted.

## MMPs and tPA-Induced Reperfusion Injury

For acute ischemic stroke, thrombolysis is beneficial for patients if induced during the first 3–4.5 h of symptom onset (NINDS, ECASS III) (National Institute of Neurological Disorders and Stroke rt-PA Stroke Study Group, 1995; Hacke et al., 2008). During this time window, thrombolysis of the occluded vessel should rescue the affected ischemic zone and improve clinical outcome. However, delayed tPA actually increases the risk of disrupting BBB integrity through changes in the cerebrovasculature. Thrombolytic tPA then can cross into the perivascular tissue and interact with the neurovascular unit (Adibhatla and Hatcher, 2008; Niego et al., 2012). Recent animal model studies of transient and permanent MCAO demonstrate that genetic deficiency or inhibition of tPA activity by neuroserpin decrease BBB disruption, edema, neuronal death, as well as improve stroke outcome (Wang et al., 1998; Tsuji et al., 2005). Furthermore, examination of both tPA KO and WT mice demonstrates that endogenous tPA is both necessary and sufficient to induce opening of the BBB after transient MCAO (Yepes et al., 2003).

The mechanism of vascular unit disruption after ischemia and reperfusion with tPA has been extensively studied. In part the disruption may result from the activation of platelet derived growth factor (PDGF)-CC, a newly discovered PDGF isoform. tPA increases BBB permeability through PDGF-CC, a known agonist of PDGF-α signaling. To determine whether MCAO-induced activation of PDGF-α regulates the cerebrovascular response to stroke, mice were treated with the PDGF-α inhibitor imatinib mesylate, also known as Gleevec or STI571, 1 h after MCAO (Su et al., 2008) (Table 2). Animals treated with imatinib exhibited a 33% reduction in Evans-Blue extravasation after MCAO compared to control mice. In addition, imatinib significantly reduced hemorrhagic complications when administered 1 h after the onset of ischemia. Treatment with neutralizing antibodies to PDGF-CC also reduced Evans-Blue extravasation.

In a separate study reported in this paper, treatment with tPA significantly increased cerebrovascular permeability, and co-injection of neutralizing antibodies to PDGF-CC with tPA blocked the increased permeability suggesting that PDGF-CC is a downstream substrate of tPA. Understanding this mechanism offers an opportunity to potentially extend the treatment window of tPA for stroke patients. Since blocking the PDGF-CC/PDGFR-α pathway is unlikely to disrupt tPA’s fibrinolytic function, strategies specifically targeting the PDGF-CC/PDGFR-α pathway should maintain tPA’s beneficial thrombolytic activity while minimizing BBB dysfunction. Further studies are needed to determine whether late administration of a combination therapy of Imatinib plus tPA can extend the standard 3 h treatment window of tPA, by reducing hemorrhagic complications and restoring neuroprotection.

Studies in experimental animal models and stroke patients strongly suggest that MMP-9 plays a central role in tPA-mediated neurotoxicity from thrombolytic therapy for acute stroke (del Zoppo, 1995; Lapchak et al., 2000; Sumii and Lo, 2002; Montaner et al., 2003; Rosell and Lo, 2008). Activated MMP-9 degrades the neurovascular matrix (Asahi et al., 2001b), and tPA amplifies total levels of MMP-9 after ischemia (Sumii and Lo, 2002; Wang et al., 2003). MMP-9 is expressed in acute ischemic stroke and upregulated by tPA in animal models. Intravenous tPA increased MMP-9 levels in ischemic brains in rats after transient MCAO (Yepes et al., 2003). Perfusion of tPA resulted in BBB disruption and degradation of the basal membrane protein, laminin, in rat blood vessels (Goto et al., 2007). In addition, MMP-9 expression, infarct size, and brain edema in tPA KO mouse were significantly lower than in WT mice, indicating that tPA can strongly increase the activity of MMP-9 in the brain (Tsuji et al., 2005).

Clinical data have also shown that plasma and brain levels of MMP-9 are elevated in patients with acute ischemic stroke, and delayed tPA therapy causes a further increase in plasma MMP-9 in stroke patients (Horstmann et al., 2003; Montaner et al., 2003; Rosell et al., 2006; Castellanos et al., 2007; Kelly et al., 2008; Rosell and Lo, 2008; Barr et al., 2010). tPA therapy independently predicted hyperacute plasma MMP-9 after adjustment for stroke severity, volume, and HT in the first 8 h after human ischemic stroke. Hyperacute MMP-9 was correlated with poor 3-month outcome (Ning et al., 2006). Thus, MMP-9 may be an important mediator of HT. Alternative thrombolytic agents or therapeutic inhibition of MMP-9 may increase the safety profile of acute stroke thrombolysis.

Several potential drugs for reducing tPA-related hemorrhagic complications have been identified. Many involve protection of the ECs, at least in part, via inhibition of MMP-9 activity (Table 2). Minocycline, a highly lipophilic semi-synthetic derivative of the tetracycline group of antibiotics that is capable of crossing the BBB, has been found to be a potent MMP inhibitor (Lee et al., 2006; Machado et al., 2006). In a rat model of embolic focal cerebral ischemia, minocycline alone effectively decreased infarct volume by about 25% (Murata et al., 2008). The combination of minocycline plus 6 h delayed tPA treatment, which by itself was not protective, reduced infarct volumes by about 50%. In addition, combination therapy with minocycline can suppress plasma levels of MMP-9 after ischemic stroke. Importantly, minocycline can lower plasma MMP-9 levels, even at 72 h after stroke, and improve neurological outcomes in acute ischemic stroke patients treated with tPA (Switzer et al., 2011). Thus, adding minocycline to delayed 6-h tPA therapy produced further benefit.

Besides its effects on infarction, minocycline may also ameliorate hemorrhage. Thrombolysis with tPA can elevate the risks of intracranial hemorrhage after ischemic stroke (National Institute of Neurological Disorders and Stroke t-PA Stroke Study Group, 1997; Larrue et al., 1999). HT occurs more frequently in patients receiving treatment at least 6 h after symptom onset (del Zoppo et al., 1992). Murata et al. (2008) determined whether minocycline can prevent tPA-associated cerebral hemorrhage and extend the reperfusion window in an experimental stroke model in rats. In this study, delayed treatment with tPA at 6 h worsened hemorrhagic conversion compared with saline and 1-h tPA; however, combining minocycline with delayed 6-h tPA decreased plasma MMP-9 levels, reduced infarction, and ameliorated brain hemorrhage. Blood levels of MMP-9 were also significantly correlated with volumes of infarction and hemorrhage. These findings suggest that minocycline may extend the therapeutic time window of tPA therapy in stroke. The translational attractiveness of this approach lies in the fact that minocycline is a relatively safe drug that can be easily used in stroke patients. Recently, it was reported in a randomized single-blinded open-label study that patients with acute ischemic stroke had significantly better outcome at day 30 and 90 with oral minocycline treatment as compared with those administered placebo (Padma Srivastava et al., 2012). These findings suggest that minocycline can be helpful in reducing the clinical deficits after acute ischemic stroke.

GM6001 is a broad-spectrum MMP inhibitor that provides protection against hemorrhagic complications induced by tPA (Table 2) (Mishiro et al., 2012). In mice subjected to 6 h MCAO with vehicle, delayed tPA alone, or combined tPA plus GM6001, GM6001 significantly reduced tPA-elevated brain hemoglobin, MMP-9 activity, and inhibited the degradation of occludin and ZO-1 induced by tPA, but not claudin-5. Treatment with GM6001 also significantly prevented the decrease in survival rate and the reduction in locomotor activity caused by tPA at 7 days after ischemia/reperfusion.

*p*-aminobenzoyl-gly-pro-d-leu-d-ala-hydroxamate (AHA) is another broad-spectrum MMP inhibitor that inhibits interstitial collagenases, gelatinases, and stromelysin with IC50 values of 1, 30, and 150 μM, respectively. AHA inhibition 3 h after MCAO in rats decreased the degree of brain edema, reduced the risk and gravity of ICH, and improved neurological outcome at 24 h (Table 2) (Copin et al., 2008). The treatment benefit was decreased if the MMP inhibition was delayed to 6 h post-ischemia, and this ameliorated the reduction in parenchymal hemorrhage. These results confirm the involvement of MMPs in HT and support the possibility of extending the window for thrombolysis in stroke by administering a broad-spectrum MMP inhibitor after ischemia.

Activated protein C (APC) is a vitamin K-dependent serine protease that inhibits blood clotting in plasma (Rezaie, 2003). It is activated from the circulating protein C zymogen on the surface of ECs. Cheng et al. (2006) reported that APC could inhibit tPA-induced hemorrhage in the ischemic rat brain (Table 2). Moreover, this effect was accompanied by inhibition of MMP-9 activity. The inhibition was absent in protease-activated receptor 1 (PAR1) KO mouse, indicating that PAR1 is required for APC-mediated down-regulation of tPA-induced MMP-9 (Cheng et al., 2006). At present, APC is not available to treat patients with ischemic stroke; however, human recombinant APC [drotrecogin alfa (activated)] was approved by the FDA for the treatment of severe sepsis or septic shock (Casserly et al., 2012). A modified APC, designed to possess significantly reduced coagulant activity (<10%) while maintaining full cytoprotective properties, is currently in preclinical development, and based upon the anticoagulant profiles, makes this new drug a feasible therapy for ischemic stroke patients (Williams et al., 2012).

Cilostazol, a selective inhibitor of phosphodiesterase III, is an antiplatelet drug and vasodilator via increased levels of cyclic AMP (cAMP) and cyclic GMP (Tanaka et al., 1989). Researchers have reported that cilostazol has a neuroprotective effect against ischemic brain injury induced by MCAO and reperfusion in rats (Choi et al., 2002; Lee et al., 2003). In addition, cilostazol prevented the HT induced by tPA and reduced the hemoglobin content and water content, as well as the MMP-9 activity in mice subjected to MCAO and reperfusion (Table 2) (Ishiguro et al., 2010). Cilostazol also prevented the decrease in claudin-5 expression, which is altered by tPA, and inhibited tPA-induced cell damage in human BMECs. This supports the idea that cilostazol limits or prevents BBB disruption after ischemic injury. The loss of claudin-5 and activation of MMP-9 were found in CD47 KO mice, indicating CD47 contributes to the disruption of BBB through activating MMP-9 (Jin et al., 2009). Thus, cilostazol could be used to downregulate the overall inflammatory MMP-9 response and upregulate claudin-5. Similar results were obtained with the Rho kinase (ROCK) inhibitor, fasudil, which was also shown to prevent MMP-9-related HT in mice treated with tPA (Table 2) (Ishiguro et al., 2012).

Matrix metalloproteinase-9 can be activated by tPA via several molecular signaling pathways, including the tPA-LRP and tPA-PAR1 pathways. tPA stimulates MMP-9 expression in brain ECs. This effect is significantly reduced in cells treated with RNAi against LRP, and was absent in LRP-deficient mouse embryonic fibroblast cells (Wang et al., 2003). Moreover, intraventricular injection of tPA into the mouse brain increased BBB permeability, although LRP antagonists blocked this effect. These findings indicate that LRP signaling pathways play an important role in tPA-induced MMP-9 activation.

In spite of the strong evidence for the role of MMP-9 in the deleterious effects of tPA, tPA toxicity could involve some other MMPs. In a recent study (Copin et al., 2011), WT and MMP-9 KO mice were subjected to 90 min transient MCAO and treated with tPA. At 24 h after occlusion, BBB permeability, hemispheric enlargement, collagen and laminin degradation, and cerebral infarction were increased in both WT and MMP-9 KO treated animals as compared with non-treated animals. Mortality was increased in WT but reduced in KO treated mice. Cerebral MMP-9 concentration was not modified by tPA. However, pre-treatment with the MMP broad-spectrum inhibitor AHA counteracted the effects of tPA on the neurovascular unit and decreased mortality in both WT and KO mice. MMP inhibition did not modify cerebral infarction in tPA-treated animals. These results suggest that tPA toxicity is mainly independent of MMP-9 after transient MCAO but could involve some other MMPs. This represents a substantial shift from the previous focus on MMP-9/BBB breakdown and the role of tPA.

Tissue plasminogen activator induces the activity of other MMPs, in addition to MMP-9. *In vitro*, tPA induces MMP-3 in cultured murine brain ECs, and this effect is prevented by inhibition of either LRP or the NF-κB activation (Suzuki et al., 2009). The involvement of the LRP/NF-κB pathway was also reported in the induction of MMP-3 by tPA under ischemic stress. In addition, the increase in both ICH and the induction of MMP-3 by tPA was suppressed by treatment with RAP, suggesting that ICH caused by tPA could be suppressed by LRP inhibition. These results support the hypothesis of dissociation between tPA-dependent mechanisms of BBB breakdown and cerebral infarction. Due to the importance of tPA in thrombolytic treatment of ischemic stroke patients, further characterization of the MMPs participating in tPA-induced BBB disruption could be clinically useful.

## Oxidative Stress and MMPs

There is a strong association between oxidative stress and MMPs in the pathophysiology of BBB damage and ischemic stroke. Oxidative stress is defined as the condition occurring when the physiological balance between oxidants and antioxidants is tipped in favor of the oxidants, increasing the risk for potential damage to the organism. Free radicals, including ROS and reactive nitrogen species (RNS), are generated soon after vessel occlusion (Lapchak et al., 2000). Accumulated free radicals not only increase the susceptibility of brain tissue to ischemic damage, but also trigger numerous molecular cascades that mediate the activation of MMPs. This leads to degradation of TJs and increased BBB permeability in the ischemic brain (Lijnen, 2001).

Elevated levels of RNS and tyrosine nitration were observed in brain sections of stroke patients and in experimental ischemic animal models (Lijnen and Collen, 1987; National Institute of Neurological Disorders and Stroke rt-PA Stroke Study Group, 1995; Miller et al., 2012; Wardlaw et al., 2012). Moreover, superoxide scavengers, nitric oxide synthase (NOS) inhibitors, and peroxynitrite decomposition catalysts attenuated BBB disruption, reduced infarction volume, and improved neurological dysfunctions in cerebral I/R injury. Superoxide dismutase (SOD2) is the principal defense against the toxicity of free radicals, which leads SOD2 KO mice to exhibit a significant increase in MMP-9 and a higher rate of brain hemorrhaging after MCAO (Maier et al., 2006). This indicates that the excess radicals can activate MMP-9, thereby inducing HT in the post-ischemic brain. These findings suggest that free radicals are an important therapeutic target for improving the outcome of an ischemic stroke.

Experimental data indicate that oxidative stress may be an early trigger of MMP upregulation after cerebral ischemia-reperfusion (Asahi et al., 2000a; Gasche et al., 2001; Liu and Rosenberg, 2005). Using human BMECs grown on porous membranes covered with BM matrix (BBB models), it was shown that ROS augmented permeability and monocyte migration across the BBB. ROS activated MMPs (MMP-1, -2, and -9) and decreased tissue inhibitors of MMPs (TIMP-1 and -2) in a protein tyrosine kinase (PTK)-dependent manner. In addition, the increase in MMPs and PTK activities paralleled degradation of BM protein and enhanced tyrosine phosphorylation of TJ protein. These effects and enhanced permeability/monocyte migration were prevented by inhibitors of MMPs or PTKs, or antioxidant therapy. The findings suggest that oxidative stress causes BBB injury via degradation of BM proteins by activated MMPs and by PTK-mediated TJ protein phosphorylation.

Reactive oxygen species and its signaling pathways can enhance tPA-mediated MMP-9 production (Harada et al., 2012). Using cultured brain ECs (b.End3) exposed to tPA, followed by H_2_O_2_, Harada et al. showed that the combination of ROS and tPA significantly increased MMP-9 production more than tPA or ROS alone. Moreover, this MMP-9 production was decreased by the addition of the free radical scavenger edaravone (MCI-186, 3-methyl-1-phenyl-2-pyrazoline-5-one), which inhibited the NF-κB pathway, specifically by enhancing I-κB degradation (Harada et al., 2012). Edaravone suppresses tPA-induced MMP-9 upregulation and hemorrhage in the transiently ischemic rat brain (Yagi et al., 2008). In a spontaneously hypertensive rat model of MCAO, administration of edaravone plus tPA increased survival rate, improved motor function, and dramatically decreased tPA-induced HT (Table 2) (Yamashita et al., 2011). In addition, edaravone suppressed MMP-9 expression at and around cerebral microvessels, inhibited the degradation of BM protein, and prevented the microvessels from dissociating. These results suggest that edaravone could protect the cerebral microvasculature because it safeguards the BM from excess free radicals and MMP-9. Thus, edaravone may provide effective neuroprotection, prevent vascular endothelial injury, and delay neuronal death in transient cerebral ischemia and ischemic stroke.

Edaravone has been used clinically in Japan and shown to be efficacious in patients with acute ischemic stroke (Suda et al., 2007) (Table 2). Edaravone also improved the functional outcome in a group of Indian patients with acute ischemic stroke (Sharma et al., 2011). In addition, administration of edaravone during tPA infusion can enhance early recanalization in acute stroke patients (Kimura et al., 2012). In a large clinical trial, another free radical scavenger, NXY-059 also reduced tPA-induced HT in humans (Table 2) (Lees et al., 2006); however, it failed to reduce disability after stroke in a subsequent trial (Shuaib et al., 2007). The clinical efficacy of NXY-059 may be limited by its reduced ability to penetrate the BBB and its restricted radical trapping capacity (Murata et al., 2008). Nevertheless, combination therapy with edaravone and tPA is a promising therapeutic strategy for acute stroke patients, not only in reducing infarct size but also in minimizing catastrophic HT.

## MMPs and Caveolin-1

Caveolae are small invaginations of the plasma membrane that form flask-like structures in many vertebrate cells, especially ECs. There are three members in the caveolin (cav) family: cav-1, 2, and 3. Cav-1 appears to be a critical determinant of BBB permeability (Gu et al., 2011). Recent studies revealed that cav-1 could prevent the degradation of TJ proteins and protect the BBB integrity by inhibiting RNS production and MMP activity. In addition, cav-1 KO mice had higher rates of apoptotic cell death and larger infarction volumes than WT mice in an experimental ischemic stroke model. Current evidence indicates that the interactions of RNS, cav-1, and MMPs are critical signal pathways in BBB disruption and infarction enlargement during cerebral ischemia-reperfusion injury (Gu et al., 2011).

Expression of cav-1 was down regulated in ischemic brains and the production of NO induced the loss of cav-1 in focal cerebral ischemia and reperfusion injury (Gu et al., 2012). The down-regulation of cav-1 was correlated with increased MMP-2 and -9 activities, decreased ZO-1 expression, and enhanced BBB permeability. Treatment with N(G) -nitro-l-arginine methyl ester (l-NAME, a non-selective NOS inhibitor) preserved the expression of cav-1, inhibited MMPs activity, and reduced BBB permeability.

To elucidate the roles of cav-1 in regulating MMPs and BBB permeability, Gu et al. (2012) used two approaches including cav-1 knock down in cultured BMECs *in vitro* and cav-1 KO mice *in vivo*. Cav-1 knock down remarkably increased MMPs activity in BMECs. Meanwhile, with focal cerebral ischemia-reperfusion, *cav-1* KO mice displayed higher MMPs activities and BBB permeability than WT mice. The effects of l-NAME on MMPs activity and BBB permeability were partly reversed in cav-1 deficient mice. These results, when taken together, suggest that cav-1 plays important roles in regulating MMPs activity and BBB permeability in focal cerebral ischemia and reperfusion injury. The effects of l-NAME on MMPs activity and BBB permeability are partly mediated by preservation of cav-1.

There is also suggestive evidence that cav-1 might at least partially regulate some endothelial TJ proteins by controlling their degradation by MMPs. Liu et al. (2012) investigated the early molecular events of ischemic BBB damage using *in vitro* oxygen-glucose deprivation (OGD) and *in vivo* rat MCAO models. Exposure of bEND3 monolayer to OGD for 2 h significantly increased its permeability to FITC-labeled dextran and promoted the secretion of MMP-2/-9 and cytosolic translocation of cav-1. This same OGD treatment also led to rapid degradation of TJ protein occludin and dissociation of claudin-5 from the cytoskeleton, which contributed to OGD-induced endothelial barrier disruption. Using the selective MMP-2/-9 inhibitor SB-3CT, their neutralizing antibodies, or cav-1 siRNA, the investigators found that MMP-2 was the major enzyme mediating OGD-induced occludin degradation, while cav-1 was responsible for claudin-5 redistribution. The interaction between cav-1 and claudin-5 was further confirmed by co-immunoprecipitation. Consistent with these *in vitro* findings, the researchers observed fluorescence tracer extravasation, increased gelatinolytic activity, and elevated interstitial MMP-2 levels in ischemic subcortical tissue after 2 h MCAO. Moreover, occludin protein loss and claudin-5 redistribution were detected in ischemic cerebromicrovessels. These data indicate that cerebral ischemia initiates two rapid parallel processes, MMP-2-mediated occludin degradation and cav-1-mediated claudin-5 redistribution, causing BBB disruption at early stroke stages. These parallel processes are relevant to the process of acute thrombolysis.

## Conclusion

Blood-brain barrier dysfunction is a critical pathophysiological process in stroke and occurs early enough to be within the thrombolytic time window. This early ischemic BBB damage is closely associated with HT and thus is emerging as a promising target for reducing the hemorrhagic complications of thrombolytic stroke therapy. However, the mechanisms underlying early ischemic BBB damage remain poorly understood. Understanding the exact role of MMPs and its signal cascades after ischemic stroke will have important diagnostic implications for stroke and for the development of therapeutic strategies aimed at modulating MMPs. Unfortunately, in view of its complex pathophysiology, which affects blood vessels, neurons, and glial cells, the quick translation of experimental discoveries into clinical practice has previously raised problems, and ischemic stroke remains one of the most challenging diseases in translational neurology. The advances in our understanding of basic stroke pathophysiology, along with improvements in the design of our basic and clinical studies, should make it possible for causative therapeutic strategies to soon find their way into clinics.

## Conflict of Interest Statement

The authors declare that the research was conducted in the absence of any commercial or financial relationships that could be construed as a potential conflict of interest.

## Abbreviations

AJ, adherens junction; APC, activated protein C; BBB, blood-brain barrier; BM, basement membrane; BMDC, bone marrow-derived cells; BMEC, brain microvascular endothelial cells; cav-1, caveolin-1; dKO, double knockout; EC, endothelial cell; ECM, extracellular matrix; HT, hemorrhagic transformation; ICH, intracranial hemorrhage; KO, knockout; l-NAME, N(G) -nitro-l-arginine methyl ester; LPS, lipopolysaccharide; LRP, lipoprotein receptor-related protein; MCAO, middle cerebral artery occlusion; MMP, metalloproteinase; NF-κB, nuclear factor kappa beta; PAR1, protease-activated receptor 1; ROS, reactive oxygen species; SB-3CT, (2-[[(4-phenoxyphenyl) sulfonyl] methyl]-thiirane); SOD2, superoxide dismutase; TIMP, tissue inhibitor of MMP; TJ, tight junction; tPA, tissue type plasminogen activator; WT, wild-type; ZO, zona occludins.

## References

[B1] Abbott N. J. Patabendige A. A. Dolman D. E. Yusof S. R. Begley D. J. (2010). Structure and function of the blood-brain barrier. Neurobiol. Dis. 37, 13–25. 10.1016/j.nbd.2009.07.030 19664713

[B2] Abbruscato T. J. Davis T. P. (1999). Protein expression of brain endothelial cell E-cadherin after hypoxia/aglycemia: influence of astrocyte contact. Brain Res. 842, 277–286. 10.1016/S0006-8993(99)01778-3 10526124

[B3] Adibhatla R. M. Hatcher J. F. (2008). Tissue plasminogen activator (tpa) and matrix metalloproteinases in the pathogenesis of stroke: therapeutic strategies. CNS Neurol. Disord. Drug Targets 7, 243–253. 10.2174/187152708784936608 18673209 PMC2562687

[B4] Asahi M. Asahi K. Wang X. Lo E. H. (2000a). Reduction of tissue plasminogen activator-induced hemorrhage and brain injury by free radical spin trapping after embolic focal cerebral ischemia in rats. J. Cereb. Blood Flow Metab. 20, 452–457. 10724108 10.1097/00004647-200003000-00002

[B5] Asahi M. Asahi K. Jung J. C. del Zoppo G. J. Fini M. E. Lo E. H. (2000b). Role for matrix metalloproteinase 9 after focal cerebral ischemia: effects of gene knockout and enzyme inhibition with BB-94. J. Cereb. Blood Flow Metab. 20, 1681–1689. 11129784 10.1097/00004647-200012000-00007

[B6] Asahi M. Wang X. Mori T. Sumii T. Jung J. C. Moskowitz M. A. (2001a). Effects of matrix metalloproteinase-9 gene knock-out on the proteolysis of blood-brain barrier and white matter components after cerebral ischemia. J. Neurosci. 21, 7724–7732. 11567062 10.1523/JNEUROSCI.21-19-07724.2001 PMC6762894

[B7] Asahi M. Sumii T. Fini M. E. Itohara S. Lo E. H. (2001b). Matrix metalloproteinase 2 gene knockout has no effect on acute brain injury after focal ischemia. Neuroreport 12, 3003–3007. 10.1097/00001756-200109170-00050 11588620

[B8] Balbin M. Fueyo A. Knauper V. Pendas A. M. Lopez J. M. Jimenez M. G. (1998). Collagenase-2 (MMP-8) expression in murine tissue-remodeling processes. Analysis of its potential role in postpartum involution of the uterus. J. Biol. Chem. 273, 23959–23968. 10.1074/jbc.273.37.23959 9727011

[B9] Balbin M. Pendas A. M. Uria J. A. Jimenez M. G. Freije J. P. Lopez-Otin C. (1999). Expression and regulation of collagenase-3 (MMP-13) in human malignant tumors. APMIS 107, 45–53. 10.1111/j.1699-0463.1999.tb01525.x 10190279

[B10] Barr T. L. Latour L. L. Lee K. Y. Schaewe T. J. Luby M. Chang G. S. (2010). Blood-brain barrier disruption in humans is independently associated with increased matrix metalloproteinase-9. Stroke 41, E123–E128. 10.1161/STROKEAHA.109.570515 20035078 PMC2827673

[B11] Bazzoni G. Dejana E. (2004). Endothelial cell to cell junctions: molecular organization and role in vascular homeostasis. Physiol. Rev. 84, 869–901. 10.1152/physrev.00035.2003 15269339

[B12] Briasoulis A. T. D. Papageorgiou N. Kampoli A. M. Androulakis E. Antoniades C. Tsiamis E. (2012). Novel therapeutic approaches targeting matrix metalloproteinases in cardiovascular disease. Curr. Top. Med. Chem. 12, 1214–1221. 10.2174/1568026611208011214 22519451

[B13] Brown S. Bernardo M. M. Li Z. H. Kotra L. P. Tanaka Y. Fridman R. (2000). Potent and selective mechanism-based inhibition of gelatinases. J. Am. Chem. Soc. 122, 6799–6800. 10.1021/ja001461n

[B14] Burrage P. S. Mix K. S. Brinckerhoff C. E. (2006). Matrix metalloproteinases: role in arthritis. Front. Biosci. 11, 529–543. 10.2741/1817 16146751

[B15] Casserly B. Gerlach H. Phillips G. S. Marshall J. C. Lemeshow S. Levy M. M. (2012). Evaluating the use of recombinant human activated protein C in adult severe sepsis: results of the Surviving Sepsis Campaign. Crit. Care Med. 40, 1417–1426. 10.1097/CCM.0b013e31823e9f45 22430247

[B16] Castellanos M. Leira R. Serena J. Pumar J. M. Lizasoain I. Castillo J. (2003). Plasma metalloproteinase-9 concentration predicts hemorrhagic transformation in acute ischemic stroke. Stroke 34, 40–46. 10.1161/01.STR.0000046764.57344.31 12511748

[B17] Castellanos M. Sobrino T. Millian M. Garcia M. Arenillas J. Nombela F. (2007). Serum cellular fibronectin and matrix metalloproteinase-9 as screening biomarkers for the prediction of parenchymal hematoma after thrombolytic therapy in acute ischemic stroke: a multifactor confirmatory study. Stroke 38, 1855–1859. 10.1161/STROKEAHA.106.481556 17478737

[B18] Chen F. Ohashi N. Li W. Eckman C. Nguyen J. H. (2009). Disruption of occludin and claudin-2 in brain endothelial cells in vitro and in brains of mice with acute liver failure. Hepatology 50, 1914–1923. 10.1002/hep.22933 19821483 PMC2925168

[B19] Chen T. Y. Lee M. Y. Chen H. Y. Kuo Y. L. Lin S. C. Wu T. S. (2006). Melatonin attenuates the postischemic increase in blood-brain barrier permeability and decreases hemorrhagic transformation of tissue-plasminogen activator therapy following ischemic stroke in mice. J. Pineal Res. 40, 242–250. 10.1111/j.1600-079X.2005.00307.x 16499561

[B20] Cheng T. Petraglia A. L. Li Z. Thiyagarajan M. Zhong Z. Wu Z. (2006). Activated protein c inhibits tissue plasminogen activator-induced brain hemorrhage. Nat. Med. 12, 1278–1285. 10.1038/nm1498 17072311

[B21] Chin J. R. Murphy G. Werb Z. (1985). Stromelysin, a connective tissue-degrading metallopeptidase secreted by stimulated rabbit synovial fibroblasts in parallel with collagenase. Biosynthesis, isolation, characterization, and substrates. J. Biol. Chem. 260, 12367–12376. 2995374

[B22] Choi J. M. Shin H. K. Kim K. Y. Lee J. H. Hong K. W. (2002). Neuroprotective effect of cilostazol against focal cerebral ischemia via antiapoptotic action in rats. J. Pharmacol. Exp. Ther. 300, 787–793. 10.1124/jpet.300.3.787 11861782

[B23] Chou S. H. Feske S. K. Simmons S. L. Konigsberg R. G. Orzell S. C. Marckmann A. (2011). Elevated peripheral neutrophils and matrix metalloproteinase 9 and biomarkers of functional outcome following subarachnoid hemorrhage. Transl. Stroke Res. 2, 600–607. 10.1007/s12975-011-0117-x 22207885 PMC3236293

[B24] Clark A. W. Krekoski C. A. Bou S. S. Chapman K. R. Edwards D. R. (1997). Increased gelatinase a (mmp-2) and gelatinase b (mmp-9) activities in human brain after focal ischemia. Neurosci. Lett. 238, 53–56. 10.1016/S0304-3940(97)00859-8 9464653

[B25] Clark W. M. Wissman S. Albers G. W. Jhamandas J. H. Madden K. P. Hamilton S. (1999). Recombinant tissue type plasminogen activator (Alteplase) for ischemic stroke 3 to 5 hours after symptom onset. The ATLANTIS Study: a randomized controlled trial. Alteplase thrombolysis for Acute noninterventional therapy in ischemic stroke. JAMA 282, 2019–2026. 10.1001/jama.282.21.2019 10591384

[B26] Copin J. C. Bengualid D. J. Da Silva R. F. Kargiotis O. Schaller K. Gasche Y. (2011). Recombinant tissue plasminogen activator induces blood-brain barrier breakdown by a matrix metalloproteinase-9-independent pathway after transient focal cerebral ischemia in mouse. Eur. J. Neurosci. 34, 1085–1092. 10.1111/j.1460-9568.2011.07843.x 21895804

[B27] Copin J. C. Merlani P. Sugawara T. Chan P. H. Gasche Y. (2008). Delayed matrix metalloproteinase inhibition reduces intracerebral hemorrhage after embolic stroke in rats. Exp. Neurol. 213, 196–201. 10.1016/j.expneurol.2008.05.022 18590727 PMC2557056

[B28] Cuadrado E. Rosell A. Penalba A. Slevin M. Alvarez-Sabin J. Ortega-Aznar A. (2009). Vascular MMP-9/TIMP-2 and neuronal MMP-10 up-regulation in human brain after stroke: a combine laser microdissection and protein array study. J. Proteome Res. 8, 3191–3197. 10.1021/pr801012x 19317417

[B151] Cucullo L. Hossain M. Puvenna V. Marchi N. Janigro D. (2011). The role of shear stress in Blood-Brain Barrier endothelial physiology. BMC Neurosci. 12:40. 10.1186/1471-2202-12-40 21569296 PMC3103473

[B29] Cui J. Chen S. Zhang C. Meng F. Wu W. Hu R. (2012). Inhibition of MMP-9 by a selective gelatinase inhibitor protects neurovasculature from embolic focal cerebral ischemia. Mol. Neurodegener. 7, 21. 10.1186/1750-1326-7-21 22587708 PMC3500265

[B30] Cunningham L. A. (2005). Multiple roles for MMPs and TIMPs in cerebral ischemia. Glia 50, 329–339. 10.1002/glia.20169 15846802

[B31] del Zoppo G. Poeck K. Pessin M. Wolpert S. Furlan A. Ferbert A. (1992). Recombinant tissue plasminogen activator in acute thrombotic and embolic stroke. Ann. Neurol. 32, 78–86. 10.1002/ana.410320113 1642475

[B32] del Zoppo G. J. (1995). Acute stroke – on the threshold of a therapy? N. Engl. J. Med. 333, 1632–1633. 10.1056/NEJM199512143332410 7477201

[B34] Demir R. Ulvi H. Ozel L. Ozdemir G. Guzelcik M. Aygul R. (2012). Relationship between plasma metalloproteinase-9 levels and volume and severity of infarct in patients with acute ischemic stroke. Acta Neurol. Belg. 112, 351–356. 10.1007/s13760-012-0067-4 22581515

[B35] Dimagl U. Iadecola C. Moskowitz M. A. (1999). Pathobiology of ischemic stroke: an integrated view. Trends Neurosci. 22, 391–397. 10.1016/S0166-2236(99)01401-0 10441299

[B36] Donnan G. A. Fisher M. MacLeod M. Davis S. M. (2008). Stroke. Lancet 371, 1612–1623. 10.1016/S0140-6736(08)60694-7 18468545

[B37] Fagan S. C. Waller J. L. Nichols F. T. Edwards D. J. Pettigrew L. C. Clark W. M. (2010). Minocycline to improve neurologic outcome in stroke (MINOS): a dose-finding study. Stroke 41, 2283–2287. 10.1161/STROKEAHA.110.582601 20705929 PMC3916214

[B38] Fernandez-Cadenas I. Del Rio-Espinola A. Carrera C. Domingues-Montanari S. Mendioroz M. Delgado P. (2012). Role of the MMP9 gene in hemorrhagic transformations after tissue-type plasminogen activator treatment in stroke patients. Stroke 43, 1398–1400. 22496335 10.1161/STROKEAHA.111.639823

[B39] Freije M. J. Diez-Itza I. Balbin M. Sanchez L. M. Blasco R. Tolivia J. (1994). Molecular cloning and expression of collagenase-3, a novel human matrix metalloproteinase produced by breast carcinoma. J. Biol. Chem. 269, 16766–16773. 8207000

[B40] Fujimoto M. Takagi Y. Aoki T. Hayase M. Marumo T. Gomi M. (2008). Tissue inhibitor of metalloproteinases protect blood-brain barrier disruption in focal cerebral ischemia. J. Cereb. Blood Flow Metab. 28, 1674–1685. 10.1038/jcbfm.2008.59 18560439

[B41] Gasche Y. Copin J. Sugawara T. Fujimura M. Chan P. K. (2001). Matrix metalloproteinase inhibition prevents oxidative stress-associated blood-brain barrier disruption after transient focal cerebral ischemia. J. Cereb. Blood Flow Metab. 21, 1393–1400. 11740200 10.1097/00004647-200112000-00003

[B42] Gasche Y. Fujimura M. Morita-Fujimura Y. Copin J. C. Kawase M. Massengale J. (1999). Early appearance of activated matrix metalloproteinase-9 after focal cerebral ischemia in mice: a possible role in blood-brain barrier dysfunction. J. Cereb. Blood Flow Metab. 19, 1020–1028. 10478654 10.1097/00004647-199909000-00010

[B43] Gidday J. M. Gasche Y. G. Copin J. C. Shah A. R. Perez R. S. Shapiro S. D. (2005). Leukocyte-derived matrix metalloproteinase-9 mediates blood-brain barrier breakdown and is proinflammatory after transient focal cerebral ischemia. Am. J. Physiol. Heart Circ. Physiol. 289, H558–H568. 10.1152/ajpheart.01275.2004 15764676

[B44] Gomez D. E. Alonso D. F. Yoshiji H. Thorgeirsson U. P. (1997). Tissue inhibitors of metalloproteinases: structure, regulation and biological function. Eur. J. Cell Biol. 74, 111–122. 9352216

[B45] Goto H. Fujisawa H. Oka F. Nomura S. Kajiwara K. Kato S. (2007). Neurotoxic effects of exogenous recombinant tissue-type plasminogen activator on the normal rat brain. J. Neurotrauma 24, 745–752. 10.1089/neu.2006.0183 17439356

[B46] Graham C. A. Chan R. W. Chan D. Y. Chan C. P. Wong L. K. Rainer T. H. (2012). Matrix metalloproteinase 9 mRNA: an early prognostic marker for patients with acute stroke. Clin. Biochem. 45, 352–355. 10.1016/j.clinbiochem.2011.12.006 22200563

[B47] Green A. R. Ashwood T. (2005). Free radical trapping as a therapeutic approach to neuroprotection in stroke: experimental and clinical studies with NXY-059 and free radical scavengers. Curr. Drug Targets CNS Neurol. Disord. 4, 109–118. 10.2174/1568007053544156 15857295

[B48] Gu Y. Dee C. M. Shen J. (2011). Interaction of free radicals, matrix metalloproteinases and caveolin-1 impact blood-brain barrier permeability. Front. Biosci. 3, 1216–1231. 10.2741/222 21622267

[B49] Gu Y. Zheng G. Xu M. Li Y. Chen X. Zhu W. (2012). Caveolin-1 regulates nitric oxide-mediated matrix metalloproteinases activity and blood-brain barrier permeability in focal cerebral ischemia and reperfusion injury. J. Neurochem. 120, 147–156. 10.1111/j.1471-4159.2011.07542.x 22007835

[B50] Gu Z. Cui J. Brown S. Fridman R. Mobashery S. Strongin A. Y. (2005). A highly specific inhibitor of matrix metalloproteinase-9 rescues laminin from proteolysis and neurons from apoptosis in transient focal cerebral ischemia. J. Neurosci. 25, 6401–6408. 10.1523/JNEUROSCI.1086-05.2005 16000631 PMC6725288

[B51] Gurney K. J. Estrada E. Y. Rosenberg G. A. (2006). Blood-brain barrier disruption by stromelysin-1 facilitates neutrophil infiltration in neuroinflammation. Neurobiol. Dis. 23, 87–96. 10.1016/j.nbd.2006.02.006 16624562

[B52] Hacke W. Kaste M. Bluhmki E. Brozman M. Davalos A. Guidetti D. (2008). Thrombolysis with alteplase 3 to 4.5 h after acute ischemic stroke. N. Engl. J. Med. 359, 1317–1329. 10.1056/NEJMoa0804656 18815396

[B53] Harada K. Suzuki Y. Yamakawa K. Kawakami J. Umemura K. (2012). Combination of reactive oxygen species and tissue-type plasminogen activator enhances the induction of gelatinase B in brain endothelial cells. Int. J. Neurosci. 122, 53–59. 10.3109/00207454.2011.623808 21919816

[B54] Harris A. K. Ergul A. Kozak A. Machado L. S. Johnson M. H. Fagan S. C. (2005). Effect of neutrophil depletion on gelatinase expression, edema formation and hemorrhagic transformation after focal ischemic stroke. BMC Neurosci. 6:49 10.1186/1471-2202-6-49 16078993 PMC1190186

[B55] Hasty K. A. Hibbs M. S. Kang A. H. Mainardi C. L. (1986). Secreted forms of human neutrophil collagenase. J. Biol. Chem. 261, 5645–5650. 3007518

[B56] Hawkins B. T. Davis T. P. (2005). The blood-brain barrier/neurovascular unit in health and disease. Pharmacol. Rev. 57, 173–185. 10.1124/pr.57.2.4 15914466

[B57] Heim-Riether A. Taylor S. J. Liang S. Gao D. A. Xiong Z. Michael A. E. (2009). Improving potency and selectivity of a new class of non-Zn-chelating MMP-13 inhibitors. Bioorg. Med. Chem. Lett. 19, 5321–5324. 10.1016/j.bmcl.2009.07.151 19692239

[B58] Herz J. Strickland D. K. (2001). LRP: a multifunctional scavenger and signaling receptor. J. Clin. Invest. 108, 779–784. 10.1172/JCI200113992 11560943 PMC200939

[B59] Horstmann S. Koziol J. Gardner H. Wagner S. (2003). Profiles of matrix metalloproteinases, their inhibitors, and laminin in stroke patients: influence of different therapies. Stroke 34, 2165–2170. 10.1161/01.STR.0000088062.86084.F2 12907822

[B60] Ishiguro M. Kawasaki K. Suzuki Y. Ishizuka F. Mishiro K. Egashira Y. (2012). A rho kinase (ROCK) inhibitor, fasudil, prevents matrix metalloproteinase-9 related hemorrhagic transformation in mice treated with tissue plasminogen activator. Neuroscience 220, 302–312. 10.1016/j.neuroscience.2012.06.015 22710066

[B61] Ishiguro M. Mishiro K. Fujiwara Y. Chen H. Izuta H. Tsuruma K. (2010). Phosphodiesterase-III inhibitor prevents hemorrhagic transformation induced by focal cerebral ischemia in mice treated with tPA. PLoS ONE 5:e15178 10.1371/journal.pone.0015178 21151895 PMC2997776

[B62] Jiang X. Namura S. Nagata I. (2001). Matrix metalloproteinase inhibitor KB-R7785 attenuates brain damage resulting from permanent focal cerebral ischemia in mice. Neurosci. Lett. 305, 41–44. 10.1016/S0304-3940(01)01800-6 11356303

[B63] Jin G. Tsuji K. Xing C. Yang T. G. Wang X. Lo E. H. (2009). CD47 gene knockout protects against transient focal cerebral ischemia in mice. Exp. Neurol. 217, 165–170. 10.1016/j.expneurol.2009.02.004 19233173 PMC3722607

[B64] Jin R. Yang G. Li G. (2010). Inflammatory mechanisms in ischemic stroke: role of inflammatory cells. J. Leukoc. Biol. 87, 779–789. 10.1189/jlb.1109766 20130219 PMC2858674

[B65] Justicia C. Panes J. Sole S. Cervera A. Deulofeu R. Chamorro A. (2003). Neutrophil infiltration increases matrix metalloproteinase-9 in the ischemic brain after occlusion/reperfusion of the middle cerebral artery in rats. J. Cereb. Blood Flow Metab. 23, 1430–1440. 14663338 10.1097/01.WCB.0000090680.07515.C8

[B66] Kelly P. J. Morrow J. D. Ning M. Koroshetz W. Lo E. H. Terry E. (2008). Oxidative stress and matrix metalloproteinase-9 in acute ischemic stroke: the Biomarker Evaluation for Antioxidant Therapies in Stroke (BEAT-Stroke) study. Stroke 39, 100–104. 10.1161/STROKEAHA.107.506212 18063832

[B67] Kim S. K. Kang S. W. Kin D. H. Yun D. H. Chung J. H. Ban J. Y. (2012). Matrix metalloproteinase-3 gene polymorphisms are associated with ischemic stroke. J. Interferon Cytokine Res. 32, 81–86. 10.1089/jir.2011.0022 22175304

[B68] Kimura K. Aoki J. Sakamoto Y. Kobayashi K. Sakai K. Inoue T. (2012). Administration of edaravone, a free radical scavenger, during t-PA infusion can enhance early recanalization in acute stroke patients – a preliminary study. J. Neurol. Sci. 313, 132–136. 10.1016/j.jns.2011.09.006 21967833

[B69] Klein T. Bischoff R. (2011). Physiology and pathophysiology of matrix metalloproteinases. Amino Acids 41, 271–290. 10.1007/s00726-010-0689-x 20640864 PMC3102199

[B70] Lapchak P. Chapman D. Zivin J. (2000). Metalloproteinase inhibition reduces thrombolytic (tissue plasminogen activator)-induced hemorrhage after thromboembolic stroke. Stroke 31, 3034–3040. 10.1161/01.STR.31.12.3034 11108768

[B71] Lapchak P. A. Araujo D. M. Song D. Wei J. Purdy R. Ja Z. (2002). Effects of the spin trap agent disodium-[(tert-butylimino) methyl] benzene-1, 3-disulfonate n-oxide (generic nxy-059) on intracerebral hemorrhage in a rabbit large clot embolic stroke model: combination studies with tissue plasminogen activator. Stroke 33, 1665–1670. 10.1161/hs0102.100530 12053009

[B72] Larrue V. Von Kummer R. Del Zoppo G. Bluhmki E. (1999). Hemorrhagic transformation in acute ischemic stroke: potential contributing factors in the ECASS study. Stroke 28, 957–960. 10.1161/01.STR.28.5.957 9158632

[B73] Lee C. Z. Xue Z. Zhu Y. Yang G. Y. Young W. L. (2007). Matrix metalloproteinase-9 inhibition attenuates vascular endothelial growth factor-induced intracerebral hemorrhage. Stroke 38, 2563–2568. 10.1161/STROKEAHA.106.481515 17673717

[B74] Lee C. Z. Yao J. S. Huang Y. Zhai W. Liu W. Guglielmo B. J. (2006). Dose-response effect of tetracyclines on cerebral matrix metalloproteinase-9 after vascular endothelial growth factor hyperstimulation. J. Cereb. Blood Flow Metab. 26, 1157–1164. 10.1038/sj.jcbfm.9600213 16395286

[B75] Lee J. H. Lee Y. K. Ishikawa M. Koga K. Fukunaga M. Miyakoda G. (2003). Cilostazol reduces brain lesion induced by focal cerebral ischemia in rats – an MRI study. Brain Res. 994, 91–98. 10.1016/j.brainres.2003.09.021 14642452

[B76] Lees K. R. Zivin J. A. Ashwood T. Davalo A. Davis S. M. Diener H. C. (2006). Stroke-Acute Ischemic NXY treatment (SAINT) Trial Investigators: Nxy-059 for acute ischemic stroke. N. Engl. J. Med. 354, 588–600. 10.1056/NEJMoa052980 16467546

[B77] Lijnen H. (2001). Plasmin and matrix metalloproteinases in vascular remodeling. Thromb. Haemost. 86, 324–333. 11487021

[B78] Lijnen H. R. Collen D. (1987). Tissue-type plasminogen activator. Ann. Biol. Clin. (Paris) 45, 198–201. 2956911

[B79] Liu J. Jin S. Liu K. J. Liu W. (2012). Matrix metalloproteinase-2-mediated occludin degradation and caveolin-1-mediated claudin-5 redistribution contribute to blood-brain barrier damage in early ischemic stroke stage. J. Neurosci. 32, 3044–3057. 10.1523/JNEUROSCI.6478-11.2012 22378877 PMC3339570

[B80] Liu K. J. Rosenberg G. (2005). Matrix metalloproteinases and free radicals in cerebral ischemia. Free Radic. Biol. Med. 39, 71–80. 10.1016/j.freeradbiomed.2005.03.033 15925279

[B81] Lo E. (2008). A new penumbra: transitioning from injury into repair after stroke. Nat. Med. 14, 497–500. 10.1038/nm1735 18463660

[B82] Lo E. H. Dalkara T. Moskowitz M. A. (2003). Mechanisms, challenges and opportunities in stroke. Nat. Rev. Neurosci. 4, 399–415. 10.1038/nrn1106 12728267

[B83] Lucivero V. Prontera M. Mezzapesa D. M. Petruzzellis M. Sancilio M. Tinelli A. (2007). Different roles of matrix metalloproteinases-2 and -9 after human ischaemic stroke. Neurol. Sci. 28, 165–170. 10.1007/s10072-007-0814-0 17690845

[B84] Machado L. S. Kozak A. Ergul A. Hess D. Borlongan C. V. Fagan S. C. (2006). Delayed minocycline inhibits ischemia-activated matrix metalloproteinases 2 and 9 after experimental stroke. BMC Neurosci. 7:56 10.1186/1471-2202-7-56 16846501 PMC1543649

[B85] Maier C. M. Hsieh L. Crandall T. Narasimhan P. Chen P. H. (2006). Evaluating therapeutic targets for reperfusion-related brain hemorrhage. Ann. Neurol. 59, 929–938. 10.1002/ana.20850 16673393

[B86] Malemud C. (2006). Matrix metalloproteinases (MMPs) in health and disease: an overview. Front. Biosci. 11, 1696–1701. 10.2741/1915 16368548

[B87] McColl B. W. Rothwell N. J. Allan S. M. (2008). Systemic inflammation alters the kinetics of cerebrovascular tight junction disruption after experimental stroke in mice. J. Neurosci. 28, 9451–9462. 10.1523/JNEUROSCI.2674-08.2008 18799677 PMC6671112

[B88] Miller J. Hartwell C. Lewandowski C. (2012). Stroke treatment using intravenous and intra-arterial tissue plasminogen activator. Curr. Treat. Options Cardiovasc. Med. 14, 273–283. 10.1007/s11936-012-0176-7 22407451

[B89] Mishiro K. Ishiguro M. Suzuki Y. Tsuruma K. Shimazawa M. Hara H. (2012). A broad-spectrum matrix metalloproteinase inhibitor prevents hemorrhagic complications induced by tissue plasminogen activator in mice. Neuroscience 205, 39–48. 10.1016/j.neuroscience.2011.12.042 22244977

[B90] Montaner J. Alvarez-Sabin J. Molina C. Angles A. Abilleira S. Arenillas J. (2001). Matrix metalloproteinase expression is related to hemorrhagic transformation after cardioembolic stroke. Stroke 32, 2762–2767. 10.1161/01.STR.32.8.1759 11739970

[B91] Montaner J. Molina C. A. Monasterio J. Abileira S. (2003). Matrix metalloproteinase pre-treatment level predicts intracranial hemorrhage complications after thrombolysis in human stroke. Circulation 107, 598–603. 10.1161/01.CIR.0000046451.38849.90 12566373

[B92] Morita K. F. M. Fujimoto K. Tsukita S. (1999). Claudin multigene family encoding four-transmembrane domain protein components of tight junction strands. Proc. Natl. Acad. Sci. U.S.A. 19, 511–516. 10.1073/pnas.96.2.511 PMC15167 9892664

[B93] Muir K. W. Tyrrell P. Sattar N. Warburton E. (2007). Inflammation and ischemic stroke. Curr. Opin. Neurol. 20, 334–342. 10.1097/WCO.0b013e32813ba151 17495630

[B94] Mun-Bryce S. Ga R. (1998). Matrix metalloproteinases in cerebrovascular disease. J. Cereb. Blood Flow Metab. 18, 1163–1172. 9809504 10.1097/00004647-199811000-00001

[B95] Mun-Bryce S. Rosenberg G. A. (1998). Matrix metalloproteinases in cerebrovascular disease. J. Cereb. Blood Flow Metab. 18, 1163–1172. 9809504 10.1097/00004647-199811000-00001

[B96] Murata Y. Rosell A. Scannevin R. H. Rhodes K. J. Wang X. Lo E. H. (2008). Extension of the thrombolytic time window with minocycline in experimental stroke. Stroke 39, 3372–3377. 10.1161/STROKEAHA.108.514026 18927459 PMC3705574

[B97] National Institute of Neurological Disorders and Stroke rt-PA Stroke Study Group. (1995). Tissue plasminogen activator for acute ischemic stroke. N. Engl. J. Med. 333, 1581–1587. 10.1056/NEJM199512143332401 7477192

[B98] National Institute of Neurological Disorders and Stroke t-PA Stroke Study Group. (1997). Intracerebral hemorrhage after intravenous t-PA therapy for ischemic stroke. Stroke 28, 2109–2118. 10.1161/01.STR.28.11.2109 9368550

[B99] Niego B. Freeman R. Puschmann T. B. Turnley A. M. Medcalf R. L. (2012). t-PA-specific modulation of a human blood-brain barrier model involves plasmin-mediated activation of the Rho kinase pathway in astrocytes. Blood 119, 4752–4761. 10.1182/blood-2011-07-369512 22262761

[B100] Ning M. Furie K. L. Koroshetz W. J. Lee H. Barron M. Lederer M. (2006). Association between tPA therapy and raised early matrix metalloproteinase-9 in acute stroke. Neurology 66, 1550–1555. 10.1212/01.wnl.0000216133.98416.b4 16717217

[B101] Nonaka Y. Tsuruma K. Shimazawa M. Yoshimura S. Iwama T. Hara H. (2009). Cilostazol protects against hemorrhagic transformation in mice transient focal cerebral ischemia-induced brain damage. Neurosci. Lett. 452, 156–161. 10.1016/j.neulet.2009.01.039 19383431

[B102] Orbe J. Barrenetxe J. Rodriguez J. A. Vivien D. Orset C. Parks W. C. (2011). Matrix metalloproteinase-10 effectively reduces infarct size in experimental stroke by enhancing fibrinolysis via a thrombin-activable fibrinolysis inhibitor-mediated mechanism. Circulation 124, 2909–2919. 10.1161/CIRCULATIONAHA.111.047100 22104553

[B103] Padma Srivastava M. V. Bhasin A. Bhatia R. Garg A. Galkwad S. Prasad K. (2012). Efficacy of minocycline in acute ischemic stroke: a single-blinded, placebo-controlled trial. Neurol. India 60, 23–28. 10.4103/0028-3886.93584 22406775

[B104] Pfefferkorn T. Rosenberg G. A. (2003). Closure of the blood-brain barrier by matrix metalloproteinase inhibition reduces mortality in cerebral ischemia with delayed reperfusion. Stroke 34, 2025–2030. 10.1161/01.STR.0000083051.93319.28 12855824

[B105] Planas A. (2001). Expression and activation of matrix metalloproteinase-2 and -9 in rat brain after transient focal cerebral ischemia. Neurobiol. Dis. 8, 834–846. 10.1006/nbdi.2001.0435 11592852

[B106] Polavarapu R. Gongora M. C. Yi H. Ranganthan S. Lawrence D. A. Strickland D. (2007). Tissue-type plasminogen activator-mediated shedding of astrocytic low-density lipoprotein receptor-related protein increases the permeability of the neurovascular unit. Blood 109, 3270–3278. 10.1182/blood-2006-08-043125 17170123 PMC1852247

[B107] Ramos-Fernandez M. Bellolio M. F. Stead L. G. (2011). Matrix metalloproteinase-9 as a marker for acute ischemic stroke: a systematic review. J. Stroke Cerebrovasc. Dis. 20, 47–54. 10.1016/j.jstrokecerebrovasdis.2009.10.008 21044610

[B108] Rezaie A. (2003). Exosite-dependent regulation of the protein C anticoagulant pathway. Trends Cardiovasc. Med. 13, 8–15. 10.1016/S1050-1738(02)00191-3 12554095

[B109] Rosell A. Alvarez-Sabin J. Arenillas J. F. Rovira A. Delgado P. Fernández-Cadenas I. (2005). A matrix metalloproteinase protein array reveals a strong relation between MMP-9 and MMP-13 with diffusion-weighted image lesion increase in human stroke. Stroke 36, 1415–1420. 10.1161/01.STR.0000170641.01047.cc 15947272

[B110] Rosell A. Lo E. H. (2008). Multiphasic roles for matrix metalloproteinases after stroke. Curr. Opin. Pharmacol. 1, 82–89. 10.1016/j.coph.2007.12.001 18226583

[B111] Rosell A. Ortega-Aznar A. Alvarez-Sabín J. Fernández-Cadenas I. Ribó M. Molina C. A. (2006). Increased brain expression of matrix metalloproteinase-9 after ischemic and hemorrhagic human stroke. Stroke 37, 1399–1406. 10.1161/01.STR.0000223001.06264.af 16690896

[B112] Rosenberg G. Estrada E. Y. Dencoff J. E. (1998). Matrix metalloproteinases and TIMPs are associated with blood-brain barrier opening after reperfusion in rat brain. Stroke 29, 2189–2195. 10.1161/01.STR.29.10.2189 9756602

[B113] Rosenberg G. A. Kornfeld M. Estrada E. Kelley R. O. Liotta L. A. Stetler-Stevenson W. G. (1992). TIMP-2 reduces proteolytic opening of blood-brain barrier by type IV collagenase. Brain Res. 576, 203–207. 10.1016/0006-8993(92)90681-X 1381261

[B114] Rosenberg G. A. Yang Y. (2007). Vasogenic edema due to tight junction disruption by matrix metalloproteinases in cerebral ischemia. Neurosurg. Focus 22, E4. 10.3171/foc.2007.22.5.5 17613235

[B115] Sakakibara A. Furuse M. Saitou M. Andro-Akatsuka Y. Tsukita S. (1997). Possible involvement of phosphorylation of occludin in tight junction formation. J. Cell Biol. 137, 1393–1401. 10.1083/jcb.137.6.1393 9182670 PMC2132539

[B116] Sandoval K. E. Witt K. A. (2008). Blood-brain barrier tight junction permeability and ischemic stroke. Neurobiol. Dis. 32, 200–219. 10.1016/j.nbd.2008.08.005 18790057

[B117] Sharma P. Sinha M. Shukla R. Garg R. K. Verma R. Singh M. K. (2011). A randomized controlled clinical trial to compare the safety and efficacy of edaravone in acute ischemic stroke. Ann. Indian Acad. Neurol. 14, 103–106. 10.4103/0972-2327.82794 21808471 PMC3141471

[B118] Shuaib A. Lees K. R. Lyden P. Grotta J. Davalos A. Davis S. M. (2007). Trial investigators: Nxy-059 for the treatment of acute ischemic stroke. N. Engl. J. Med. 357, 562–571. 10.1056/NEJMoa070240 17687131

[B119] Sole S. Petegnief V. Gorina R. Chamorro A. Planas A. M. (2004). Activation of matrix metalloproteinase-3 and agrin cleavage in cerebral ischemia/reperfusion. J. Neuropathol. Exp. Neurol. 63, 338–349. 15099024 10.1093/jnen/63.4.338

[B120] Sopata I. Dancewicz A. M. (1974). Presence of a gelatin-specific proteinase and its latent form in human leucocytes. Biochim. Biophys. Acta 370, 510–523. 10.1016/0005-2744(74)90112-0 4216367

[B121] Staddon J. M. Herrenknecht K. Smales C. Rubin L. L. (1995). Evidence that tyrosine phosphorylation may increase tight junction permeability. J. Cell. Sci. 108, 609–619. 7769005 10.1242/jcs.108.2.609

[B122] Sternlicht M. D. Werb Z. (2001). How matrix metalloproteinases regulate cell behavior. Annu. Rev. Cell Dev. Biol. 17, 463–516. 10.1146/annurev.cellbio.17.1.463 11687497 PMC2792593

[B123] Stuart R. O. Nigam S. K. (1995). Regulated assembly of tight junctions by protein kinase C. Proc. Natl. Acad. Sci. U.S.A. 92, 6072–6076. 10.1073/pnas.92.13.6072 7597083 PMC41644

[B124] Su E. J. Fredriksson L. Geyer M. Folestad E. Cale J. Andrae J. (2008). Activation of pdgf-cc by tissue plasminogen activator impairs blood-brain barrier integrity during ischemic stroke. Nat. Med. 14, 731–737. 10.1038/nm1787 18568034 PMC2811427

[B125] Suda S. Igarashi H. Arai Y. Andou J. Chishiki T. Katayama Y. (2007). Effect of edaravone, a free radical scavenger on ischemic cerebral edema assessed by magnetic resonance imaging. Neurol. Med. Chir. (Tokyo) 47, 197–201. 10.2176/nmc.47.197 17527045

[B126] Sumii T. Lo E. (2002). Involvement of matrix metalloproteinase in thrombolysis-associated hemorrhagic transformation after embolic focal ischemia is rats. Stroke 33, 831–836. 10.1161/hs0302.104542 11872911

[B127] Suofu Y. Clark J. F. Broderick J. P. Kurosawa Y. Wagner K. R. Lu A. (2012). Matrix metalloproteinase-2 or -9 deletions protect against hemorrhagic transformation during early stage of cerebral ischemia and reperfusion. Neuroscience 212, 180–189. 10.1016/j.neuroscience.2012.03.036 22521821 PMC3367043

[B128] Suzuki Y. Nagai N. Umemura K. Collen D. Lijnen H. R. (2007). Stromelysin-1 (MMP-3) is critical for intracranial bleeding after t-PA treatment of stroke in mice. J. Thromb. Haemost. 5, 1732–1739. 10.1111/j.1538-7836.2007.02628.x 17596135

[B129] Suzuki Y. Nagai N. Yamakawa K. Kawakami J. Lijnen H. R. Umemura K. (2009). Tissue-type plasminogen activator (t-PA) induced stromelysin-1 (MMP-3) in endothelial cells through activation of lipoprotein receptor-related protein. Blood 114, 3352–3358. 10.1182/blood-2009-02-203919 19608750

[B130] Switzer J. A. Hess D. C. Ergul A. Waller J. L. Machado L. S. Portik-Dobos V. (2011). Matrix metalloproteinase-9 in an exploratory trial of intravenous minocycline for acute ischemic stroke. Stroke 42, 2633–2635. 10.1161/STROKEAHA.111.633008 21737808 PMC3181080

[B131] Taddei A. Giampietro C. Conti A. Orsenigo F. Breviario F. Pirazzoli V. (2008). Endothelial adherens junctions control tight junctions by VE-cadherin-mediated upregulation of claudin-5. Nat. Cell Biol. 10, 923–934. 10.1038/ncb1752 18604199

[B132] Tanaka K. Gotoh F. Fukuuchi Y. Amano T. Uematsu D. Kawamura J. (1989). Effects of a selective inhibitor of cyclic AMP phosphodiesterase on the pial microcirculation in feline cerebral ischemia. Stroke 20, 668–673. 10.1161/01.STR.20.5.668 2718208

[B133] Tejima E. Guo S. Murata Y. Arai K. Lok J. Van Leyen K. (2009). Neuroprotective effects of overexpressing tissue inhibitor of metalloproteinase TIMP-1. J. Neurotrauma 26, 1935–1941. 10.1089/neu.2009.0959 19469687 PMC2822804

[B134] Tsuji K. Aoki T. Tejima E. Arai K. Lee S. R. Atochin D. N. (2005). Tissue plasminogen activator promotes matrix metalloproteinase-9 upregulation after focal cerebral ischemia. Stroke 36, 1954–1959. 10.1161/01.STR.0000177517.01203.eb 16051896

[B135] Vincent P. A. Xiao K. Buckley K. M. Kowalczyk A. P. (2004). VE-cadherin: adhesion at arm’s length. Am. J. Physiol. Cell Physiol. 286, C987–C997. 10.1152/ajpcell.00522.2003 15075197

[B136] Wang G. Guo Q. Hossain M. Fazio V. Zeynalov E. Janigro D. (2009). Bone marrow-derived cells are the major source of MMP-9 contributing to blood-brain barrier dysfunction and infarct formation after ischemic stroke in mice. Brain Res. 1294, 183–192. 10.1016/j.brainres.2009.07.070 19646426 PMC2758551

[B137] Wang X. Lee S. R. Arai K. Lee S. R. Tsuji K. Reveck G. W. (2003). Lipoprotein receptor-mediated induction of matrix metalloproteinase by tissue plasminogen activator. Nat. Med. 9, 1313–1317. 10.1038/nm926 12960961

[B138] Wang Y. F. Tsirka S. E. Strickland S. Stieg P. E. Soriano S. G. Lipton S. A. (1998). Tissue plasminogen activator (tPA) increases neuronal damage after focal cerebral ischemia in wild-type and tPA-deficient mice. Nat. Med. 4, 228–231. 10.1038/nm0298-228 9461198

[B139] Wardlaw J. M. Murray V. Berge E. Del Zoppo G. Sandercock P. Lindley R. L. (2012). Recombinant tissue plasminogen activator for acute ischaemic stroke: an updated systematic review and meta-analysis. Lancet 379, 2364–2372. 10.1016/S0140-6736(12)60738-7 22632907 PMC3386494

[B140] Williams P. D. Zlokovic B. V. Griffin J. H. Pryor K. E. Davis T. P. (2012). Preclinical safety and pharmacokinetic profile of 3K3A-APC, a novel modified activated protein C for ischemic stroke. Curr. Pharm. Des. 18, 4215–4222. 10.2174/138161212802430413 22632606 PMC3472038

[B141] Yagi K. Kitazato K. T. Uno M. Tada Y. Kinouchi T. Shimada K. (2008). Edaravone, a free radical scavenger inhibits MMP-9-related brain hemorrhage in rats treated with tissue plasminogen activator. Stroke 40, 626–631. 10.1161/STROKEAHA.108.520262 19095969

[B142] Yamamoto M. Ramirez S. H. Sato S. Kiyota T. Cerny R. L. Kaibuchi K. (2008). Phosphorylation of claudin-5 and occludin by rho kinase in brain endothelial cells. Am. J. Pathol. 172, 521–533. 10.2353/ajpath.2008.070076 18187566 PMC2312373

[B143] Yamashita T. Abe K. (2011). Therapeutic approaches to vascular protection in ischemic stroke. Acta Med. Okayama 65, 219–223. 21860527 10.18926/AMO/46846

[B144] Yamashita T. Deguchi K. Nagotani S. Abe K. (2011). Vascular protection and restorative therapy in ischemic stroke. Cell Transplant. 20, 95–97. 10.3727/096368910X539092 20887680

[B145] Yamashita T. Kamiya T. Deguchi K. Inaba T. Zhang H. Shang J. (2009). Dissociation and protection of the neurovascular unit after thrombolysis and reperfusion in ischemic rat brain. J. Cereb. Blood Flow Metab. 29, 715–725. 10.1038/jcbfm.2008.164 19142198

[B146] Yang Y. Estrada E. Y. Thompson J. F. Liu W. Rosenberg G. A. (2007). Matrix metalloproteinase-mediated disruption of tight junction proteins in cerebral vessels is reversed by synthetic matrix metalloproteinase inhibitor in focal ischemia in rat. J. Cereb. Blood Flow Metab. 27, 697–709. 10.1038/sj.jcbfm.9600440 16850029

[B147] Yang Y. Rosenberg G. A. (2011). MMP-mediated disruption of claudin-5 in the blood-brain barrier of rat brain after cerebral ischemia. Methods Mol. Biol. 762, 333–345. 10.1007/978-1-61779-185-7_24 21717368 PMC4950933

[B148] Yepes M. Sandkvist M. Moore E. G. Bugge T. H. Strickland D. K. Lawrence D. A. (2003). Tissue-type plasminogen activator induces opening of the blood-brain barrier via the LDL receptor-related protein. J. Clin. Invest. 112, 1533–1540. 10.1172/JCI19212 14617754 PMC259131

[B149] Zhao B. Q. Wang S. Kim H.-Y. Storrie H. Rosen B. R. Mooney D. J. (2006). Role of matrix metalloproteinases in delayed cortical responses after stroke. Nat. Med. 12, 441–445. 10.1038/nm1387 16565723

[B150] Zlokovic B. V. (2006). Remodeling after stroke. Nat. Med. 12, 390–391. 10.1038/nm0406-390 16598283

