# Corrigendum: Matrix Metalloproteinases and Blood-Brain Barrier Disruption in Acute Ischemic Stroke

**DOI:** 10.3389/fneur.2018.00202

**Published:** 2018-04-04

**Authors:** Shaheen E. Lakhan, Annette Kirchgessner, Deborah Tepper, Aidan Leonard

**Affiliations:** ^1^Biosciences Department, Global Neuroscience Initiative Foundation, Beverly Hills, CA, United States; ^2^Neurological Institute, Cleveland Clinic, Cleveland, OH, United States; ^3^School of Health and Medical Sciences, Seton Hall University, South Orange, NJ, United States

**Keywords:** matrix metalloproteinases, blood-brain barrier, stroke, caveolin-1, reactive oxygen species

**Missing Citation 1**

In the original article, Cucullo et al. (2011) was not cited in the article. The citation has now been inserted in “Structural Components of the BBB/Neurovascular Unit,” Paragraph 1 and should read:

The BBB is a dynamic interface between the peripheral circulation and the CNS. It controls the influx and efflux of biological substances needed for the brain metabolic processes, as well as for neuronal function. Thus, the functional and structural integrity of the BBB is vital in maintaining brain homeostasis (Cucullo et al., 2011).

**Missing Citation 2**

In the original article, Klein and Bischoff (2011) was cited in the article except for a single instance. The citation has now been inserted in “MMP-9 (Gelatinase B),” Paragraph 1 and should read:

Matrix metalloproteinase-9 (gelatinase B), first described in neutrophils in 1974 (Sopata and Dancewicz, 1974), is expressed as a 92-kDa proenzyme, which can be activated to the 83-kDa mature enzyme (Klein and Bischoff, 2011).

**Missing Citation 3**

In the original article, Malemud (2006) was cited in the article except for a single instance. The citation has now been inserted in “Matrix Metalloproteinases,” Paragraph 1 and should read:

Most of the MMPs are synthesized as inactive latent enzymes. Conversion to the active enzyme is generally mediated by activator systems that include plasminogen activator or the pro-hormone convertase, furin (Malemud, 2006).

**Text Correction**

In the original article, there was an error. An incorrect citation was made to del Zoppo (2009) instead of Hawkins and Davis (2005).

A correction has been made to “Structural Components of the BBB/Neurovascular Unit,” Paragraph 2:

Briefly, the anatomical substrate of the BBB is the cerebral microvascular endothelium, which together with the closely associated astrocytes, pericytes, neurons, and the ECM, constitute a “neurovascular unit” that is essential for the health and function of the CNS (Hawkins and Davis, 2005).

The authors apologize for these errors and state that these do no change the scientific conclusions of the article in any way.

The original article has been updated.

## Conflict of Interest Statement

The authors declare that the research was conducted in the absence of any commercial or financial relationships that could be construed as a potential conflict of interest.
